# Supplementary material for: Patients’ experiences with fluctuations in persistent physical symptoms: a qualitative study
Source: BMJ Open. 2020 Jul 13;10(7):e035833. doi: 10.1136/bmjopen-2019-035833 (PMC7359057; doi:10.1136/bmjopen-2019-035833)
Supplement: Author's manuscript [file bmjopen-2019-035833.draft_revisions.pdf]

BMJ Open is committed to open peer review. As part of this commitment we make the peer review history of every article we publish publicly available.

When an article is published we post the peer reviewers' comments and the authors' responses online. We also post the versions of the paper that were used during peer review. These are the versions that the peer review comments apply to.

The versions of the paper that follow are the versions that were submitted during the peer review process. They are not the versions of record or the final published versions. They should not be cited or distributed as the published version of this manuscript.

BMJ Open is an open access journal and the full, final, typeset and author-corrected version of record of the manuscript is available on our site with no access controls, subscription charges or pay-per-view fees (http://bmjopen.bmj.com).

If you have any questions on BMJ Open's open peer review process please email info.bmjopen@bmj.com

BMJ Open

Fluctuations in persistent physical symptoms: a patient perspective

Journal:	*BMJ Open*
Manuscript ID	bmjopen-2019-035833
Article Type:	Original research
Date Submitted by the Author:	18-Nov-2019
Complete List of Authors:	Barends, Hieke; Amsterdam UMC - Locatie VUMC, General Practice and Elderly Care Medicine; Amsterdam Public Health research institute<br>Walstock, Ella; Amsterdam UMC - Locatie VUMC, Department of General Practice and Elderly Care Medicine; Amsterdam Public Health research institute<br>Botman, Femke; Amsterdam UMC - Locatie VUMC, Department of General Practice and Elderly Care Medicine; Amsterdam Public Health research institute<br>de Kruif , Anja ; Vrije Universiteit Amsterdam, Department of Health Sciences, Faculty of Science; Amsterdam Public Health research institute<br>Claassen, Nikki; Amsterdam UMC - Locatie VUMC, Department of General Practice and Elderly Care Medicine; Amsterdam Public Health research institute<br>van der Wouden, Johannes; Amsterdam UMC - Locatie VUMC, Department of General Practice and Elderly Care Medicine; Amsterdam Public Health research institute<br>Olde Hartman, Tim; Radboud University Nijmegen Medical Center, Department of Primary and Community Care; Radboud University Donders Institute for Brain Cognition and Behaviour<br>Dekker, Joost; Amsterdam UMC - Locatie VUMC, Department of Rehabilitation Medicine and Department of Psychiatry; Amsterdam Public Health research institute<br>van der Horst, Henriette; Amsterdam UMC - Locatie VUMC, Department of General Practice and Elderly Care Medicine; Amsterdam Public Health research institute
Keywords:	QUALITATIVE RESEARCH, PRIMARY CARE, GENERAL MEDICINE (see Internal Medicine)

SCHOLARONE™
Manuscripts

I, the Submitting Author has the right to grant and does grant on behalf of all authors of the Work (as defined in the below author licence), an exclusive licence and/or a non-exclusive licence for contributions from authors who are: i) UK Crown employees; ii) where BMJ has agreed a CC-BY licence shall apply, and/or iii) in accordance with the terms applicable for US Federal Government officers or employees acting as part of their official duties; on a worldwide, perpetual, irrevocable, royalty-free basis to BMJ Publishing Group Ltd ("BMJ") its licensees and where the relevant Journal is co-owned by BMJ to the co-owners of the Journal, to publish the Work in this journal and any other BMJ products and to exploit all rights, as set out in our licence.

The Submitting Author accepts and understands that any supply made under these terms is made by BMJ to the Submitting Author unless you are acting as an employee on behalf of your employer or a postgraduate student of an affiliated institution which is paying any applicable article publishing charge ("APC") for Open Access articles. Where the Submitting Author wishes to make the Work available on an Open Access basis (and intends to pay the relevant APC), the terms of reuse of such Open Access shall be governed by a Creative Commons licence – details of these licences and which Creative Commons licence will apply to this Work are set out in our licence referred to above.

Other than as permitted in any relevant BMJ Author's Self Archiving Policies, I confirm this Work has not been accepted for publication elsewhere, is not being considered for publication elsewhere and does not duplicate material already published. I confirm all authors consent to publication of this Work and authorise the granting of this licence.

For peer review only

Fluctuations in persistent physical symptoms: a patient perspective

Hieke Barends, MD[1,2]*
Ella Walstock, MSc, medical student[1,2]
Femke Botman, MSc, medical anthropologist[1,2]
Anja (JThCM) de Kruif, MSc, health scientist, senior researcher[3]
Nikki Claassen- van Dessel, MD, PhD, general practitioner[1,2]
Johannes C. van der Wouden, PhD, sociologist and associate professor[1,2]
Tim olde Hartman, MD, PhD, general practitioner, senior researcher[5]
Joost Dekker, PhD, psychologist, professor of Allied Health Care [2,4]
Henriëtte E. van der Horst, MD, PhD, general practitioner, professor of General Practice[1,2]

[1] Amsterdam UMC, Vrije Universiteit Amsterdam, Department of General Practice and Elderly Care Medicine, the Netherlands
[2] Amsterdam Public Health research institute
[3] Department of Health Sciences, Faculty of Science, Amsterdam Public Health Institute, Vrije Universiteit Amsterdam, the Netherlands
[4] Amsterdam UMC, Vrije Universiteit Amsterdam, Department of Rehabilitation Medicine and Department of Psychiatry, the Netherlands
[5] Department of Primary and Community Care, Donders Institute for Brain, Cognition and Behaviour, Radboud University Nijmegen Medical Center, Nijmegen, the Netherlands

For peer review only

***Corresponding author:**
Hieke Barends
Amsterdam UMC, Vrije Universiteit Amsterdam
Department of General Practice and Elderly Care Medicine
Amsterdam Public Health research institute,
Medical Faculty, room C-378
Van der Boechorststraat 7, 1081 BT Amsterdam
Tel: +31 20-4449678
Email: h.barends@amsterdamumc.nl

Word count: 3991 words

Email of all other authors: e.r.walstock@student.rug.nl; f.botman@amsterdamumc.nl; anja.de.kruif@vu.nl; nclaassen.vandessel@gmail.com; j.vanderwouden@amsterdamumc.nl; Tim.OldeHartman@radboudumc.nl; j.dekker@amsterdamumc.nl; he.vanderhorst@amsterdamumc.nl

For peer review only - http://bmjopen.bmj.com/site/about/guidelines.xhtml

Abstract

Objectives: To explore patients' experiences with fluctuations in persistent physical symptoms (PPS) and to understand which factors -from their viewpoint- play a role in these fluctuations.

Design: Qualitative study using semi-structured interviews and thematic content analysis.

Setting: This qualitative study is part of a multi-center prospective cohort study on the course of PPS. Patients were recruited in general practices and specialized treatment facilities for PPS throughout the Netherlands.

Participants: Interviews were conducted with a sample of fifteen patients with PPS to explore their experiences with fluctuations in symptom severity.

Results: We identified three themes in the analysis: (1) experiences with symptom fluctuations (2) physical limits and emotional boundaries and (3) dealing with fluctuations: experienced difficulties in balancing limits and boundaries. Daily and weekly fluctuations in symptoms were an important element in patients' experiences. Patients searched for explanations in order to prevent symptoms from worsening over the day and week. Worsening in symptoms and stronger fluctuations were experienced when overstepping physical limits and/or crossing emotional boundaries. Patients experienced difficulties in respecting these limits and boundaries. An important impeding factor was the lack of recognition of symptoms in the absence of a diagnosis or plausible explanation. Without a diagnosis, patients often felt left alone in dealing with their symptoms and limitations. According to them, resignation and taking personal limits and boundaries seriously resulted in a better balance, less extreme fluctuations and improved wellbeing.

Conclusions: Dealing with fluctuations in the severity of symptoms is an important element of the symptom experience in patients with PPS and poses various challenges. Resignation and taking physical limits and emotional boundaries seriously are experienced as critical steps towards less fluctuations and improved wellbeing.

Keywords

Patients experiences
Fluctuations in symptoms
Qualitative study
Persistent Physical Symptoms
Medically Unexplained Symptoms

For peer review only

Article Summary

Strengths and limitations of this study

- Qualitative research was applied to understand patients' experiences with fluctuations of symptoms and factors playing a role in these fluctuations from their perspective.
- Our study highlights that dealing with fluctuations is an important element of the symptom experience in PPS and deserves more attention in care for these patients and in research.
- Patients were recruited in general practices as well as in specialized PPS programs in different parts of the Netherlands, and in that regard represent a broad sample of patients with persistent physical symptoms.
- All of the recruited patients experienced (episodes of) severe persistent physical symptoms and most experienced symptoms for an extensive period of time (>5 years), therefore our findings may be less applicable to patients experiencing mild or moderate symptoms or symptoms of short duration.

For peer review only - http://bmjopen.bmj.com/site/about/guidelines.xhtml

Introduction

Patients with physical symptoms not attributable to verifiable, conventionally defined diseases are common in all medical settings. These symptoms are often referred to as "medically unexplained symptoms". A recent and perhaps more appropriate term -putting less emphasis on the mind-body dualism in the origin of symptoms- is persistent physical symptoms (PPS)(1, 2). When these symptoms persist, they can have a severe impact on patients' quality of life and functional capabilities, and also on society due to high medical care utilization and loss of productivity (3, 4).

Fluctuations in symptoms have been described in several functional somatic syndromes (FSS) such as Chronic Fatigue Syndrome (CFS) and fibromyalgia (5-7), which all fall under the umbrella of PPS. Most studies on the course of PPS in a broad sample of patients used a single follow-up measurement in time to determine improvement or deterioration. According to a number of studies conducted in primary and secondary health care settings, 50-75% of patients showed symptom improvement over time, whereas 10-30% worsened (8). In a cohort study on the course of PPS we found improvement (63%) and deterioration (27%) rates were in line with prior literature, when using total changes scores based on two measurements (9). However, when four available measurements were taken into account, the temporal stability of these outcomes was limited, as intra-patient fluctuations were highly prevalent.

To the best of our knowledge, no prior qualitative study focused specifically on fluctuations of symptoms in PPS. Having a better idea of the experiences of these fluctuations may be helpful for medical professionals who provide care for these patients. This knowledge may enable them to understand what their patients are dealing with and to provide better guidance and support to patients with PPS. Therefore, the aims of this qualitative study were to explore patients' experiences with fluctuations in the severity of symptoms and to gain insight into factors influencing fluctuations in their symptoms from the patients' perspective.

Methods

Study design

The present study was part of a larger prospective cohort study that monitors the course of symptoms and physical functioning in patients with PPS. We chose a qualitative design and conducted semi-structured (in-depth) interviews, to obtain information about the experiences of patients with PPS. The institutional review board of the Amsterdam UMC (IRB00002991) approved the research protocol (No. 2018.483). Patients or the public were not actively involved in the design, conduct, reporting or dissemination plans of our research.

Participants

Participants were selected from the PROSPECTS study (see Box 1). For the PROSPECTS study, patients filled in questionnaires about the nature and severity of their symptoms (PHQ-15, 0-30 scale (10)) and physical functioning (RAND-36 PCS, 0-100 scale (11)) among other questionnaires. We wanted to include patients with fluctuations as well as patients with a (seemingly) stable course of their PPS, because symptom experiences in terms of stability and fluctuations might differ between these patients.

For peer review only - http://bmjopen.bmj.com/site/about/guidelines.xhtml

Therefore, we selected patients who: (1) showed either clinically relevant fluctuations or clinical stability (based on minimal clinically important differences) in symptom severity (PHQ-15) and physical functioning (RAND-36 PCS) over a three-year time period and (2) had given informed consent to be contacted for future research. We used purposive sampling to ensure a diversity of participants in terms of nature of symptoms, age, gender, social characteristics (educational level, living in a rural/ urban area) and recruitment setting.

Patients were approached by phone by HB or EW. In total, 21 patients were contacted. Two patients were not willing to participate because of personal reasons, three patients refused because of time constraints. One patient cancelled the interview appointment due to work-related reasons. All selected patients provided written informed consent.

Fifteen patients agreed to participate. All of the recruited patients experienced (episodes of) severe persistent physical symptoms and most experienced symptoms for an extensive period of time (>5 years). Nature of symptoms varied. Almost all of them (N=14) had symptoms in at least two symptom clusters and a substantial number (N=10) in at least three symptom clusters. Other characteristics of the patients are shown in table 1.

For peer review only

For peer review only - http://bmjopen.bmj.com/site/about/guidelines.xhtml

Box 1. The PROSPECTS study

The PROSPECTS study is a Dutch longitudinal cohort study following patients (N=325) with persistent physical symptoms (PPS). PPS patients aged between 18-70 years were recruited in general practices (N=218) and in specialized PPS programs of secondary and tertiary care organizations (N=107) across the Netherlands in 2013-2015. Initially patients were followed over a period of three years with five measurements in time (baseline, 6, 12, 24, 36 months of follow-up) (van Dessel et al 2014). In 2017, the follow-up period was extended to a period of five years, adding a 48 and 60 months follow-up measurement. Baseline characteristics and information on the recruitment process and first two years of follow-up have been published elsewhere (9, 12).

Definition of PPS: PPS was defined as the presence of physical symptoms, which had lasted at least several weeks and for which no sufficient explanation was found after proper medical examination by a physician. This is in line with the current Dutch multidisciplinary and general practice guidelines for Medically Unexplained Symptoms (MUS) (13,14).

peer review only

Table 1. Patient characteristics

Variable	n/15
**Fluctuations/ stability**	
-Fluctuations in SS and PF	9/15
-Stable in SS and PF	5/15
-Fluctuations in SS, stable in PF	1/15
**Mean age (years, range)**	55.4 years (range 32-73 years)
**Gender**	
-male	3
-female	12
**Education**	
-higher educational level	4
-intermediate educational level	4
-lower educational level	7
**Living area**	
-rural area	5
-city	10
**Recruitment setting**	
-general practice	12
-specialized PPS program	3

SS: symptom severity (based on minimal clinically important differences in PHQ-15)
PF: physical functioning (based on minimal clinically important differences in RAND-36 PCS)

For peer review only - http://bmjopen.bmj.com/site/about/guidelines.xhtml

Data collection

Interviews took place between January and April 2019. Based on the preference of the patient, eleven interviews were conducted at the patients' home and four at the research department of the university in a private meeting room. All interviews were digitally recorded. The interviews took 60 minutes on average (range: 33 -93 minutes). Patients received a gift voucher of € 15,-. Participants were told that the main interviewer (HB) is a GP registrar and researcher with an interest in PPS and the fellow interviewer (EW) a medical intern involved in a research project on PPS. Both interviewers are female. HB had received training in qualitative research and was supervised by an experienced qualitative researcher (AdK).

Interviews were loosely structured using a topic guide with relevant areas explored in depth. The main interviewer (HB) emphasized that that all interviews were non-judgmental, confidential and anonymized. She also told the participants the researchers were particularly interested in the course of their symptoms over shorter (days, weeks) and longer (months, years) periods of time. The topic guide consisted of five main topics: (1) the experienced course of symptoms and how symptoms interfered with their daily activities, with special focus on stability and fluctuations over time (day, week, month, year(s)); (2) factors contributing to fluctuations in symptoms; (3) management of symptoms and fluctuations (4) the role of their social and work environment; (5) the role of the healthcare system and care providers. Patients were encouraged to talk freely about their experiences and expand on any aspects they felt were relevant. The topic guide was checked throughout the interview process, no major adjustments were made. All participants received a summary of the interview afterwards for a member check. Fourteen patients responded to the summaries, they confirmed that they recognized their experiences in the summaries and no major changes in content were made.

Data analysis

All interviews were transcribed verbatim and coded using Atlas.ti version 7. The analyzing process was based on thematic analysis according to the six phases described by Braun and Clarke (15). In all phases at least two authors were involved (HB, EW, FB) to increase reliability. In the first phase, HB, EW and FB familiarized themselves with the data by summarizing and close reading. In the second phase, HB, EW and FB all read and coded the first two interview transcripts, using open coding. Codes were discussed to reach agreement and to improve reliability. This resulted in an initial code list that was extended when further transcripts were analyzed in pairs following the same strategy. In the following phases, codes were clustered into sub-themes in order to identify patterns in the interviews, after which final themes were identified. Codes, sub-themes and themes were discussed by HB, EW and FB until consensus was reached on all themes. Constant comparison was used in order to understand differences and similarities between patients and within each patient. All results were discussed in the research team to enhance the robustness of the findings. Finally, the report was produced and quotes were extracted that related to the themes. We used the SRQR checklist when writing our report (16).

For peer review only - http://bmjopen.bmj.com/site/about/guidelines.xhtml

Results

Three main themes were prominent in the analysis: (1) experiences with symptom fluctuations (2) physical limits and emotional boundaries and (3) dealing with fluctuations: experienced difficulties in respecting limits and setting boundaries.

1. Experiences with symptom fluctuations

All of the interviewed patients described fluctuations in the occurrence and severity of symptoms as an important element of the symptoms they experienced, none of the patients experienced symptoms as stable in severity. Fluctuations in symptoms occurred in particular over the day, but also over the week.

"And it varies. One day I am in the shower and I think 'here it comes'. The next day, well, it can start during the day. And sometimes, very occasionally, I will be fine." (P4, female, 47 yrs)

Most patients experienced a gradual worsening of symptoms over the day and work week. Worsening over the work week was described by all patients with (un)paid jobs.

"If I wake up with little pain, it is a good day. But a day will eventually always end with pain." (P3, female, 32 yrs)

"At the end of the week it is usually worse." (P8, male, 62 yrs)

Only few patients did not experience a recognizable pattern over the day or week. Most patients also described exacerbations and remissions of symptoms and how these symptoms influenced their lives over longer periods of time (months-years).

"And I've also had periods when I was able to do other things as well. So there have been periods when things were better, and I could do a little more." (P2, male, 56 yrs)

Throughout their lives, a couple of patients described several isolated episodes of symptom exacerbations that lasted at least several months, as well as periods that had been free of symptoms. At the time of the interview, a couple of participants reported a recent increase in symptoms over the weeks before the interview, whereas one patient was free of symptoms at the time of the interview. In some patients improvement was present, but only for relatively short periods.

"Well, yes, there are bad days and good days, but then there are more bad ones." (P12, female, 70 yrs)

In particular fluctuations over the day and week resulted in a continuous search to understand and explain what caused the worsening of symptoms in order to anticipate on and prevent symptoms from worsening. Responding and anticipating to worsening of symptoms impacted their daily planning and routines. Many patients expressed that to prevent worsening of symptoms, they needed to adjust their daily activities to their possibilities and take enough rest in between activities so symptoms could decrease in intensity.

"After being active and moving around for about twenty minutes I need to sit down. So I clean the toilet, then sit down and have a cup of coffee and do some reading. And after that I, for example, take out the laundry." (P1, female, 55 yrs)

"The entire day I keep in mind what I need and want to do. So if I have a birthday tonight, than I take a nap in the afternoon." (P13, male, 41 yrs)

For peer review only - http://bmjopen.bmj.com/site/about/guidelines.xhtml

2. Physical limits and emotional boundaries

A central theme in relation to the experienced fluctuations was personal limits and boundaries. Participants unanimously described an increase in symptoms when overstepping their physical limits. Some patients also mentioned an increase in symptoms when crossing their emotional boundaries. A number of patients also explicitly mentioned these factors as important explanations for the experienced fluctuations in symptoms. They indicated it often was after a long personal process – mostly taking several years- to establish the connection between worsening of symptoms and overstepping personal limits and boundaries.

Balancing physical limits

"You see, if I don't take part in any strenuous activities, every day will be more or less the same. But as soon as I engage in some strenuous activities, it will change and I will have one or two days with more severe symptoms." (P2, male, 56 yrs)

Overdoing it resulted in setbacks with worse symptoms and stronger fluctuations. This resulted in a search for balance: a balance between their aims and abilities, pushing physical limits but not overdoing it.

"At first I was up and down, all over the place. I really thought 'I'll get over this, I'll do it again, I'll do everything again (…) Well, it takes a couple of years before you really hit the wall and think 'sorry, you can try as hard as you like, you will still have these setbacks.' And then you can start all over again, because then you are overstepping your limits." (P11, female, 54 yrs)

Some patients described an energy balance. In case of a negative balance, symptoms worsened. Many patients experienced a link between this energy balance and the progression of their symptoms during the day or week.

"You know, it's like 'everybody has an energy span, a range of ability, that is different for every person, and you always want something else'. Only I am usually just confronted with the consequences of this sooner. Because when I think 'I'll keep going now', I'll have a problem tomorrow." (P3, female, 32 yrs)

Almost all patients mentioned the importance of respecting their physical limits in order to prevent their symptoms from worsening and to experience fewer fluctuations. Some patients also mentioned the importance of staying active and searching for the right balance, as *not doing enough* also resulted in worse symptoms in these patients.

"It is 'I did either too much, or not enough'. One or the other." (P11, female, 54 yrs)

Crossing emotional boundaries

A number of patients mentioned not just the importance of respecting their physical limitations, but also the importance of respecting their emotional boundaries.

"And sometimes I think: I am not going to do that. If I am not well and it's not something I really enjoy. No … I evaluate: is it worthwhile, does it do me any good? Is it something I enjoy? If not, I say no. You also need to learn to say 'No'. I didn't do that when this started." (P12, female, 70 yrs)

A couple of patients indicated that their symptoms also worsened following acute emotionally stressful events. In these cases they felt not capable to control symptoms.

For peer review only - http://bmjopen.bmj.com/site/about/guidelines.xhtml

"In an event like that, I won't be able to sit down. There is too much adrenaline in my system. A whole lot of symptoms will follow. Not directly, but after a day or two, when things are calming down a bit." (P3, female, 32 yrs)

One patient linked her symptoms solely to negative emotions. She found the solution in respecting her emotional boundaries and changing her personal situation with help of her religion. She was free of symptoms at the time of the interview.

"Well, you know, you are angry, you are sad, or a little depressed. (…) Why is this happening to me? (…) But the physical pains I sometimes had, that was purely because I was sad. You know, that stress that sometimes enters your system. And it also has to do with resignation. How much of your situation do you accept?" (P5, female, 55 yrs)

3. Dealing with fluctuations: experienced difficulties in balancing limits and boundaries

Patients described issues that were either helpful or hampering in balancing their physical limits and respecting their emotional boundaries. An important impeding factor was the lack of recognition of symptoms in the absence of a diagnosis or plausible explanation. Considered helpful was resigning to their condition and taking their personal limits and boundaries serious.

Lack of recognition and validation of symptoms

Patients described that not having their symptoms recognized and confirmed by a diagnosis or plausible explanation made them push past their physical limits over and over again, resulting in a worsening of symptoms and fluctuations in severity. It caused difficulties in respecting their physical limits. It contributed to feelings of failure and frustration, not being able to keep up with others, nor to be able to do the same things as before the symptoms started.

"Only, realistically, I sometimes think 'well, but I don't have a problem'. 'There is nothing wrong with me'. Everything is in working order, so I should be able to just do that. Often this is what gets in the way, like 'it's all in my mind'. You know, why not push through? But then I immediately pay the price." (P3, female, 32 yrs)

In learning how to live and deal with their symptoms and limits, they felt rarely supported by health care providers (HCP). Instead, their symptoms were often denied by HCP.

"I used to have a GP who said 'Well, but there is nothing wrong with you.' But then I think: if only you could experience it for a day… because it is not 'nothing'. It is just not true that 'nothing is wrong'." (P3, female, 32 yrs)

Patients frequently felt left alone in dealing with their symptoms and limitations, in case of absence of a respected label or medical diagnosis.

"So at a certain point the GP doesn't know what to do anymore; he examines you and says: 'go see a physiotherapist or a manual therapist'. And if they can't help either, and they tell you 'well then it must be in your mind', then you are left empty-handed." (P13, male, 41 yrs)

The unexplained and often invisible nature of their symptoms and limitations also made it hard to find recognition by employers, relatives and friends. This resulted in difficulties in setting and maintaining their limits and boundaries when they had to deal with others. In particular employed patients struggled with this, as they felt they had to push their limits continuously and struggled to be taken serious.

For peer review only - http://bmjopen.bmj.com/site/about/guidelines.xhtml

Importance of resigning to limits and boundaries

Many patients described that realizing they had to resign themselves to their condition and take their limits and boundaries seriously was helpful in the process towards improved wellbeing. It resulted in a better balance and less fluctuations in symptoms. Several patients described this as a moment of 'flipping the switch': from searching for an explanation and cure for symptoms towards resignation of symptoms and limits and changing focus to improving their wellbeing. They described this as a deeply personal process, in which you have to take the lead yourself.

"So it's just a switch you have to flip. And if you don't, then you'll never get anywhere. Because it is all up to you." (P1, female, 55 yrs)

Although many patients mentioned the importance of resigning to personal limits and boundaries, they described this process as challenging. The uncertainty and usually long duration of the diagnostic process and hope for a diagnosis and cure was mentioned by many as hampering the process towards resignation of symptoms and taking their limitations seriously. Finding a plausible explanation for the experienced symptoms, was viewed as helpful in resigning to personal limits and boundaries. Most patients – with or without an explanation- eventually adjusted their daily planning to their limits and possibilities. They mentioned pacing activities, respecting their physical limits and resting effectively as important strategies in gaining control over symptoms, experiencing fewer ups and downs. Respecting their emotional boundaries by learning to say 'no' was mentioned by some as helpful. Consciously deciding to participate in activities they knew would result in worsening of symptoms, valuing an activity as 'worth it', helped some patients to cope. These strategies were learned by a few patients in psychotherapy.

"Yes, and also that you know when you are overdoing it and you still choose to do that, knowing that you will be in serious pain the next day. That makes it easier to accept. The harder you fight, the angrier, I think, you will get and the worse your pain will be." (P11, female, 54 yrs)

Discussion

Our findings highlight that dealing with fluctuations in symptoms, in particular over the day and week, is an important element of symptom experience in patients with PPS. It impacts their daily routines and poses various challenges. Patients experience worsening of symptoms and stronger fluctuations when overstepping physical limits and/ or when emotional boundaries were crossed. The lack of recognition of symptoms in the absence of a diagnosis was experienced as an impeding factor in taking their personal limits and boundaries serious. Without a diagnosis, patients often felt left alone in dealing with their condition and experienced a lack of advice on how to cope with their symptoms and limits. Resignation and taking limits and boundaries seriously are experienced as critical steps towards less fluctuations, a better balance and contributed to their wellbeing.

As far as we are aware, this is the first qualitative study exploring the experiences with fluctuations in symptoms among patients with PPS. A strength of this study was the fact that patients were recruited in different health care settings throughout the Netherlands and that patients varied with regard to diversity of symptoms and demographic and social characteristics. More female patients were interviewed, but numbers were in line with the balance in the cohort from which we selected the patients (75% female). A limitation of our study, is that all interviewed patients experienced (episodes of) severe PPS for longer periods of time and our findings may be less applicable to patients experiencing mild or moderate symptoms or symptoms of short duration. Another limitation is that the interviewed patients are a sample of patients willing to participate in a study on PPS, which might have caused selection bias.

For peer review only - http://bmjopen.bmj.com/site/about/guidelines.xhtml

Our findings correspond to some findings from quantitative studies in CFS. Ecologic momentary assessment established that patients experienced difficulties in balancing their activities in response to symptoms. More fatigue related symptoms and pain predicted more activity limitation whereas feeling subjectively well predicted more all-or-nothing behavior, resulting in ups and downs (5). Pacing activities was helpful in preventing fluctuations in symptoms (6). Comparable quantitative studies in a broader sample of patients with PPS are currently lacking. Our findings, however, suggest that dealing with fluctuations and experiencing difficulties in balancing limits and boundaries seem to apply to the broader spectrum of PPS.

In our study, patients indicated that letting go of the search for a diagnosis and cure and resigning to their symptoms and limits, was an important step towards experiencing less fluctuations and improved wellbeing. A prior qualitative study described resignation and acceptance of PPS as a stage that usually took place when patients understood that finding a diagnosis and/or cure for their condition was unlikely. This study by Kornelson et al. described that in particular patients who displayed acceptance –as opposed to resignation- shifted their focus towards improving their quality of life (17). In our study most patients indicated that even though they did not fully accept their symptoms and kept struggling with their limitations, they were able to resign to their limits and shift their focus towards improving their wellbeing. So, resignation and acceptance seem closely related, but the latter implies to be a later stage in a process of change. Resignation and/ or acceptance may be a precondition to change focus to improving wellbeing: acceptance was an important condition for symptom improvement (18) and facilitated a process of change towards self-compassion and self-care in patients with PPS (19).

Several of our findings may be helpful in the care for patients with PPS. First, our study again underlines the need to take symptoms and their consequences seriously as a HCP, also in the absence of an identifiable disease. Patients with PPS face particular challenges in the resignation of symptoms, as a respected label or diagnosis is lacking. Secondly, as a HCP, exploring patients' experiences with their physical limits and/or emotional boundaries might be a useful starting point to gain an understanding of what your patient is struggling with on a daily basis and may create a common ground for supportive care to improve wellbeing and provide illness-based interventions and advice. Most patients we interviewed felt left alone in dealing with their symptoms and limits in the absence of a diagnosis and struggled to find recognition of their condition, which hampered resignation and acceptance of limits and boundaries.

Our study highlights that fluctuations in symptoms are an important element of the experienced symptoms. More longitudinal research into short-term fluctuations in experienced symptoms in a broad sample of patients with PPS, for example by the experience sampling method (ESM), could provide useful new insights. ESM can reveal how symptom experience relates to implicit patterns of thought, experience and behavior (20). Another valuable area of research could be the process towards resignation in the absence of a diagnosis. A better understanding and more knowledge of how caregivers can facilitate the process towards resignation might be helpful, in particular because resigning to limits and boundaries –although experienced as crucial in improving wellbeing – appears challenging in the absence of an accepted diagnostic label.

Acknowledgements

We want to thank all the patients that took part in this study for sharing their experiences.

Funding

For peer review only - http://bmjopen.bmj.com/site/about/guidelines.xhtml

This work was supported by grants of ZonMw [funding number 839110018] and the Stoffels-Hornstra Foundation. The funding sources had no role in the design, analyses and interpretation of this study.

Author Statement

HB, NCvD, JCvdW, JD and HvdH developed the study protocol. AdK and ToH provided feedback on the study protocol. HB and EW contributed to the development of the topic guide, collected and analysed the data and interpreted the results. FB analysed the data and interpreted the results. AdK provided feedback on the data collection and analysis. HB drafted the manuscript. All authors read, provided critical revisions and approved the manuscript.

Competing interests

Apart from the institutional grants mentioned above, there are no other potential competing interests to report.

Data sharing statement

No additional data are available.

References

1. Picariello F, Ali S, Moss-Morris R, Chalder T. The most popular terms for medically unexplained symptoms: the views of CFS patients. J Psychosom Res. 2015;78(5):420-6.
2. Marks EM, Hunter MS. Medically Unexplained Symptoms: an acceptable term? Br J Pain. 2015;9(2):109-14.
3. Konnopka A, Schaefert R, Heinrich S, Kaufmann C, Luppa M, Herzog W, et al. Economics of medically unexplained symptoms: a systematic review of the literature. Psychother Psychosom. 2012;81(5):265-75.
4. Rask MT, Ornbol E, Rosendal M, Fink P. Long-Term Outcome of Bodily Distress Syndrome in Primary Care: A Follow-Up Study on Health Care Costs, Work Disability, and Self-Rated Health. Psychosom Med. 2017;79(3):345-57.
5. Band R, Barrowclough C, Caldwell K, Emsley R, Wearden A. Activity patterns in response to symptoms in patients being treated for chronic fatigue syndrome: An experience sampling methodology study. Health Psychol. 2017;36(3):264-9.
6. Meeus M, van Eupen I, van Baarle E, De Boeck V, Luyckx A, Kos D, et al. Symptom fluctuations and daily physical activity in patients with chronic fatigue syndrome: a case-control study. Arch Phys Med Rehabil. 2011;92(11):1820-6.
7. Zautra AJ, Fasman R, Parish BP, Davis MC. Daily fatigue in women with osteoarthritis, rheumatoid arthritis, and fibromyalgia. Pain. 2007;128(1-2):128-35.
8. olde Hartman TC, Borghuis MS, Lucassen PL, van de Laar FA, Speckens AE, van Weel C. Medically unexplained symptoms, somatisation disorder and hypochondriasis: course and prognosis. A systematic review. J Psychosom Res. 2009;66(5):363-77.
9. Claassen-van Dessel N, van der Wouden JC, Hoekstra T, Dekker J, van der Horst HE. The 2-year course of Medically Unexplained Physical Symptoms (MUPS) in terms of symptom severity and functional status: results of the PROSPECTS cohort study. J Psychosom Res. 2018;104:76-87.
10. Kroenke K, Spitzer RL, Williams JB. The PHQ-15: validity of a new measure for evaluating the severity of somatic symptoms. Psychosom Med. 2002;64(2):258-66.
11. VanderZee KI, Sanderman R, Heyink JW, de Haes H. Psychometric qualities of the RAND 36-Item Health Survey 1.0: a multidimensional measure of general health status. Int J Behav Med. 1996;3(2):104-22.

For peer review only - http://bmjopen.bmj.com/site/about/guidelines.xhtml

12. Claassen-van Dessel N, van der Wouden JC, Dekker J, van der Horst HE. Clinical value of DSM IV and DSM 5 criteria for diagnosing the most prevalent somatoform disorders in patients with medically unexplained physical symptoms (MUPS). J Psychosom Res. 2016;82:4-10.
13. Feltz-Cornelis vdCM, Swinkels JA, Blankenstein AH, Hoedeman R, Keuter EJ. De Nederlandse multidisciplinaire richtlijn 'Somatisch onvoldoende verklaarde lichamelijke klachten en somatoforme stoornissen'. Nederlands Tijdschrift voor Geneeskunde. 2011;155(18).
14. Olde Hartman T, Blankenstein AH, Molenaar B, Bentz van den Berg B, van der Horst HE, Arnold I, et al. NHG Standaard Somatisch onvoldoende verklaarde lichamelijke klachten (SOLK). Huisarts en Wetenschap. 2013;56(5):222-30.
Kornelsen J, Atkins C, Brownell K, Woollard R. The Meaning of Patient Experiences of Medically Unexplained Physical Symptoms. Qual Health Res. 2016;26(3):367-76.
15. Braun V, Clarke V. Using thematic analysis in psychology. Qual Res Psychol 2006;3:77–101.
16. O'Brien BC, Harris IB, Beckman TJ, Reed DA, Cook DA. Standards for reporting qualitative research: a synthesis of recommendations. Acad Med. 2014;89(9):1245-51
17. Kornelsen J, Atkins C, Brownell K, Woollard R. The Meaning of Patient Experiences of Medically Unexplained Physical Symptoms. Qual Health Res. 2016;26(3):367-76.
18. Claassen-van Dessel N, R. Velzeboer F, C. van der Wouden J, den Boer C, Dekker J, E. van der Horst H. Patients' Perspectives On Improvement Of Medically Unexplained Physical Symptoms: a Qualitative Analysis. Mental Health in Family Medicine. 2015;11: 42-48.
19. van Ravesteijn HJ, Suijkerbuijk YB, Langbroek JA, Muskens E, Lucassen PL, van Weel C, et al. Mindfulness-based cognitive therapy (MBCT) for patients with medically unexplained symptoms: process of change. J Psychosom Res. 2014;77(1):27-33.
20. van Os Jim J. The experience sampling method as an mHealth tool to support self-monitoring, self-insight, and personalized health care in clinical practice. Depression Anxiety. 2017;34(6):481-93.

For peer review only - http://bmjopen.bmj.com/site/about/guidelines.xhtml

Reporting checklist for qualitative study.

Based on the SRQR guidelines.

Instructions to authors

Complete this checklist by entering the page numbers from your manuscript where readers will find each of the items listed below.

Your article may not currently address all the items on the checklist. Please modify your text to include the missing information. If you are certain that an item does not apply, please write "n/a" and provide a short explanation.

Upload your completed checklist as an extra file when you submit to a journal.

In your methods section, say that you used the SRQRreporting guidelines, and cite them as:

O'Brien BC, Harris IB, Beckman TJ, Reed DA, Cook DA. Standards for reporting qualitative research: a synthesis of recommendations. Acad Med. 2014;89(9):1245-1251.

		Reporting Item	Page Number
**Title**			
	#1	Concise description of the nature and topic of the study identifying the study as qualitative or indicating the approach (e.g. ethnography, grounded theory) or data collection methods (e.g. interview, focus group) is recommended	4
**Abstract**			
	#2	Summary of the key elements of the study using the abstract format of the intended publication; typically includes background, purpose, methods, results and conclusions	1
**Introduction**			
Problem formulation	#3	Description and signifcance of the problem / phenomenon studied: review of relevant theory and empirical work; problem statement	4

For peer review only

Purpose or research question	#4	Purpose of the study and specific objectives or questions	4
**Methods**			
Qualitative approach and research paradigm	#5	Qualitative approach (e.g. ethnography, grounded theory, case study, phenomenolgy, narrative research) and guiding theory if appropriate; identifying the research paradigm (e.g. postpositivist, constructivist / interpretivist) is also recommended; rationale. The rationale should briefly discuss the justification for choosing that theory, approach, method or technique rather than other options available; the assumptions and limitations implicit in those choices and how those choices influence study conclusions and transferability. As appropriate the rationale for several items might be discussed together.	4,7
Researcher characteristics and reflexivity	#6	Researchers' characteristics that may influence the research, including personal attributes, qualifications / experience, relationship with participants, assumptions and / or presuppositions; potential or actual interaction between researchers' characteristics and the research questions, approach, methods, results and / or transferability	7
Context	#7	Setting / site and salient contextual factors; rationale	4,6,7
Sampling strategy	#8	How and why research participants, documents, or events were selected; criteria for deciding when no further sampling was necessary (e.g. sampling saturation); rationale	4,5
Ethical issues pertaining to human subjects	#9	Documentation of approval by an appropriate ethics review board and participant consent, or explanation for lack thereof; other confidentiality and data security issues	4
Data collection methods	#10	Types of data collected; details of data collection procedures including (as appropriate) start and stop dates of data collection and analysis, iterative	7

For peer review only

		process, triangulation of sources / methods, and modification of procedures in response to evolving study findings; rationale	
Data collection instruments and technologies	#11	Description of instruments (e.g. interview guides, questionnaires) and devices (e.g. audio recorders) used for data collection; if / how the instruments(s) changed over the course of the study	7
Units of study	#12	Number and relevant characteristics of participants, documents, or events included in the study; level of participation (could be reported in results)	5,6
Data processing	#13	Methods for processing data prior to and during analysis, including transcription, data entry, data management and security, verification of data integrity, data coding, and anonymisation / deidentification of excerpts	7
Data analysis	#14	Process by which inferences, themes, etc. were identified and developed, including the researchers involved in data analysis; usually references a specific paradigm or approach; rationale	7
Techniques to enhance trustworthiness	#15	Techniques to enhance trustworthiness and credibility of data analysis (e.g. member checking, audit trail, triangulation); rationale	7
**Results/findings**			
Syntheses and interpretation	#16	Main findings (e.g. interpretations, inferences, and themes); might include development of a theory or model, or integration with prior research or theory	8,9,10,11
Links to empirical data	#17	Evidence (e.g. quotes, field notes, text excerpts, photographs) to substantiate analytic findings	8,9,10,11
**Discussion**			
Intergration with prior work, implications, transferability and contribution(s) to the field	#18	Short summary of main findings; explanation of how findings and conclusions connect to, support, elaborate on, or challenge conclusions of earlier scholarship; discussion of scope of application /	11,12

For peer review only

		generalizability; identification of unique contributions(s) to scholarship in a discipline or field	
Limitations	#19	Trustworthiness and limitations of findings	11
**Other**			
Conflicts of interest	#20	Potential sources of influence of perceived influence on study conduct and conclusions; how these were managed	13
Funding	#21	Sources of funding and other support; role of funders in data collection, interpretation and reporting	12

The SRQR checklist is distributed with permission of Wolters Kluwer © 2014 by the Association of American Medical Colleges. This checklist was completed on 15. November 2019 using https://www.goodreports.org/, a tool made by the EQUATOR Network in collaboration with Penelope.ai

For peer review only

BMJ Open

Patients' experiences with fluctuations in persistent physical symptoms: a qualitative study

Journal:	*BMJ Open*
Manuscript ID	bmjopen-2019-035833.R1
Article Type:	Original research
Date Submitted by the Author:	15-Apr-2020
Complete List of Authors:	Barends, Hieke; Amsterdam UMC - VUMC location, General Practice and Elderly Care Medicine; Amsterdam Public Health research insitute<br>Walstock, Ella; Amsterdam UMC - VUMC location, Department of General Practice and Elderly Care Medicine; Amsterdam Public Health research institute<br>Botman, Femke; Amsterdam UMC - VUMC location, Department of General Practice and Elderly Care Medicine; Amsterdam Public Health research institute<br>de Kruif , Anja ; Vrije Universiteit Amsterdam, Department of Health Sciences, Faculty of Science<br>Claassen, Nikki; Amsterdam UMC - VUMC location, Department of General Practice and Elderly Care Medicine; Amsterdam Public Health research institute<br>van der Wouden, Johannes; Amsterdam UMC - VUMC location, Department of General Practice and Elderly Care Medicine; Amsterdam Public Health research institute<br>Olde Hartman, Tim; Radboud University Nijmegen Medical Center, Department of Primary and Community Care; Radboud University Donders Institute for Brain Cognition and Behaviour<br>Dekker, Joost; Amsterdam UMC - VUMC location, Department of Rehabilitation Medicine and Department of Psychiatry; Amsterdam Public Health research institute<br>van der Horst, Henriette; Amsterdam UMC - VUMC location, Department of General Practice and Elderly Care Medicine; Amsterdam Public Health research institute
**Primary Subject Heading**:	Qualitative research
Secondary Subject Heading:	General practice / Family practice, Patient-centred medicine
Keywords:	QUALITATIVE RESEARCH, PRIMARY CARE, GENERAL MEDICINE (see Internal Medicine)

SCHOLARONE™
Manuscripts

For peer review only - http://bmjopen.bmj.com/site/about/guidelines.xhtml

I, the Submitting Author has the right to grant and does grant on behalf of all authors of the Work (as defined in the below author licence), an exclusive licence and/or a non-exclusive licence for contributions from authors who are: i) UK Crown employees; ii) where BMJ has agreed a CC-BY licence shall apply, and/or iii) in accordance with the terms applicable for US Federal Government officers or employees acting as part of their official duties; on a worldwide, perpetual, irrevocable, royalty-free basis to BMJ Publishing Group Ltd ("BMJ") its licensees and where the relevant Journal is co-owned by BMJ to the co-owners of the Journal, to publish the Work in this journal and any other BMJ products and to exploit all rights, as set out in our licence.

The Submitting Author accepts and understands that any supply made under these terms is made by BMJ to the Submitting Author unless you are acting as an employee on behalf of your employer or a postgraduate student of an affiliated institution which is paying any applicable article publishing charge ("APC") for Open Access articles. Where the Submitting Author wishes to make the Work available on an Open Access basis (and intends to pay the relevant APC), the terms of reuse of such Open Access shall be governed by a Creative Commons licence – details of these licences and which Creative Commons licence will apply to this Work are set out in our licence referred to above.

Other than as permitted in any relevant BMJ Author's Self Archiving Policies, I confirm this Work has not been accepted for publication elsewhere, is not being considered for publication elsewhere and does not duplicate material already published. I confirm all authors consent to publication of this Work and authorise the granting of this licence.

For peer review only

Patients' experiences with fluctuations in persistent physical symptoms: a qualitative study

Hieke Barends, MD[1,2]*
Ella Walstock, MSc, medical student[1,2]
Femke Botman, MSc, medical anthropologist[1,2]
Anja (JThCM) de Kruif, MSc, health scientist, senior researcher[3]
Nikki Claassen- van Dessel, MD, PhD, general practitioner[1,2]
Johannes C. van der Wouden, PhD, sociologist and associate professor[1,2]
Tim olde Hartman, MD, PhD, general practitioner, senior researcher[5]
Joost Dekker, PhD, psychologist, professor of Allied Health Care [2,4]
Henriëtte E. van der Horst, MD, PhD, general practitioner, professor of General Practice[1,2]

[1] Amsterdam UMC, Vrije Universiteit Amsterdam, Department of General Practice and Elderly Care Medicine, the Netherlands
[2] Amsterdam Public Health research institute
[3] Department of Health Sciences, Faculty of Science, Amsterdam Public Health Institute, Vrije Universiteit Amsterdam, the Netherlands
[4] Amsterdam UMC, Vrije Universiteit Amsterdam, Department of Rehabilitation Medicine and Department of Psychiatry, the Netherlands
[5] Department of Primary and Community Care, Donders Institute for Brain, Cognition and Behaviour, Radboud University Nijmegen Medical Center, Nijmegen, the Netherlands

Corresponding author:
Hieke Barends
Amsterdam UMC, Vrije Universiteit Amsterdam
Department of General Practice and Elderly Care Medicine
Amsterdam Public Health research institute,
Medical Faculty, room C-378
Van der Boechorststraat 7, 1081 BT Amsterdam
Tel: +31 20-4449678
Email: h.barends@amsterdamumc.nl

Word count: 4324 words

Email of all other authors: ellawalstock@gmail.com; f.botman@amsterdamumc.nl; anja.de.kruif@vu.nl; nclaassen.vandessel@gmail.com; j.vanderwouden@amsterdamumc.nl; Tim.OldeHartman@radboudumc.nl; j.dekker@amsterdamumc.nl; he.vanderhorst@amsterdamumc.nl

For peer review only - http://bmjopen.bmj.com/site/about/guidelines.xhtml

Abstract

Objectives: To explore patients' experiences with fluctuations in persistent physical symptoms (PPS) and to understand which factors -from their viewpoint- play a role in these fluctuations.

Design: Qualitative study using semi-structured interviews and thematic content analysis.

Setting: This qualitative study is part of a multi-center prospective cohort study on the course of PPS. Patients were recruited in general practices and specialized treatment facilities for PPS throughout the Netherlands.

Participants: Interviews were conducted with a sample of fifteen patients with PPS to explore their experiences with fluctuations in symptom severity.

Results: We identified three themes in the analysis: (1) Patterns in symptom fluctuations (2) Perceived causes of symptom exacerbations, and (3) Patients' strategies in gaining control over symptom exacerbations. Daily and weekly fluctuations in symptoms were an important element in patients' experiences. In particular anticipating on the worsening of symptoms impacted their daily routines and posed various challenges. Symptom exacerbations were attributed to overstepping physical limits and/or the impact of negative emotions. Resigning to physical limits, adjusting ones daily planning, weighing personal needs and learning to say 'no' were described as different strategies in gaining control over symptom exacerbations.

Conclusions: Fluctuations in the severity of symptoms -and in particular daily and weekly symptom exacerbations- are an important element of the symptom experience in patients with PPS and poses various challenges. Patients attributed symptom exacerbation to overstepping physical limits and/or negative emotions. Patients described different strategies in gaining control over symptom exacerbations.

Keywords

Patients experiences
Fluctuations in symptoms
Qualitative study
Persistent Physical Symptoms
Medically Unexplained Symptoms

For peer review only

For peer review only - http://bmjopen.bmj.com/site/about/guidelines.xhtml

Article Summary

Strengths and limitations of this study

- Qualitative research was applied to understand patients' experiences with fluctuations of symptoms and factors playing a role in these fluctuations from their perspective.
- Our study highlights that fluctuations in the experienced severity of symptoms -and in particular daily and weekly symptom exacerbations- are an important element of the symptom experience in PPS and deserve more attention in care for these patients and in research.
- Patients were recruited in general practices as well as in specialized PPS programs in different parts of the Netherlands, and in that regard represent a broad sample of patients with persistent physical symptoms.
- All of the recruited patients experienced (episodes of) severe PPS and most experienced symptoms for an extensive period of time (>5 years), therefore our findings may be less applicable to patients experiencing mild or moderate symptoms or symptoms of short duration.

For peer review only

For peer review only - http://bmjopen.bmj.com/site/about/guidelines.xhtml

Introduction

Patients with physical symptoms not attributable to verifiable, conventionally defined diseases are common in all medical settings. These symptoms are often referred to as “medically unexplained symptoms” (MUS). A recent and perhaps more appropriate term -putting less emphasis on the mind-body dualism in the origin of symptoms- is persistent physical symptoms (PPS) (1, 2). When these symptoms persist, they can have a severe impact on patients’ quality of life and functional capabilities and also on society due to high medical care utilization and loss of productivity (3, 4).

There has been extensive debate about definitions and terminology in this field of research. Whereas some emphasize commonalities and overlap in symptoms and characteristics (5-8), others differentiate between particular functional somatic syndromes (FSS), such as fibromyalgia, chronic fatigue syndrome and irritable bowel syndrome (9-11). The importance of studying both similarities as well as differences has also been highlighted (12). In this study we focus on similarities and overlap in patients’ symptom experiences. We defined PPS as symptoms, which last at least several weeks and for which no sufficient somatic explanation is found after proper medical examination by a physician. This is in line with the current Dutch multidisciplinary and general practice guidelines for MUS (PPS) (13, 14). So, by definition our umbrella term PPS may also cover several FSS.

Fluctuations in symptoms have been described in several quantitative studies in patients with FFS (15-17). Most studies on the course of PPS in a broad sample of patients used a single follow-up measurement in time to determine improvement or deterioration. According to a number of studies conducted in primary and secondary health care settings, 50-75% of patients with PPS showed symptom improvement over time, whereas 10-30% worsened (18). In a cohort study that we conducted on the course of PPS we found improvement (63%) and deterioration (27%) rates that were in line with prior literature, when using total changes scores based on two measurements. However, when four available measurements were taken into account, the temporal stability of these outcomes was limited, as intra-patient fluctuations were highly prevalent (19). These findings suggest that most patients with PPS might experience exacerbations and remissions in symptoms.

To the best of our knowledge, no prior qualitative study focused specifically on fluctuations of symptoms in PPS. Understanding the experiences of fluctuations in symptom severity may help medical professionals in providing care for these patients. This knowledge may enable them to understand what their patients are dealing with and to provide better guidance and support to patients with PPS. Therefore, the aims of this qualitative study were to explore patients’ experiences with fluctuations in the severity of symptoms and -if present- to gain insight into factors influencing fluctuations in their symptoms from the patients’ perspective.

Methods

Study design

The present study was part of a larger prospective cohort study that monitors the course of symptoms and physical functioning in patients with PPS. We chose a qualitative design and conducted semi-structured (in-depth) interviews, to obtain information about the experiences of patients with PPS. The institutional review board of the Amsterdam UMC (IRB00002991) approved the research protocol (No. 2018.483).

Patient and public involvement

Patients or the public were not actively involved in the design, conduct, reporting or dissemination plans of our research.

For peer review only - http://bmjopen.bmj.com/site/about/guidelines.xhtml

Participants

Participants were selected from the PROSPECTS study (see Box 1). For the PROSPECTS study, patients filled in questionnaires about the nature and severity of their symptoms (PHQ-15, 0-30 scale (20)) and physical functioning (RAND-36 PCS, 0-100 scale (21)) among other questionnaires. We wanted to include patients with fluctuations as well as patients with a (seemingly) stable course of their PPS, because symptom experiences in terms of stability and fluctuations might differ between these patients. Therefore, we selected patients who: (1) showed either clinically relevant fluctuations or clinical stability (based on minimal clinically important differences) in symptom severity (PHQ-15) and physical functioning (RAND-36 PCS) over a three-year time period and (2) had given informed consent to be contacted for future research. We used purposive sampling to ensure a diversity of participants in terms of nature of symptoms, age, gender, social characteristics (educational level, living in a rural/ urban area) and recruitment setting. Over a three years' period, only a minority of the participants (<15%) showed clinical stability in symptom severity and physical functioning.

Patients were approached by phone by HB or EW. In total, 21 patients were contacted. Two patients were not willing to participate because of personal reasons, three patients refused because of time constraints. One patient cancelled the interview appointment due to work-related reasons. All selected patients provided written informed consent.

Fifteen patients agreed to participate. All of the recruited patients experienced (episodes of) severe PPS and most experienced symptoms for an extensive period of time (>5 years). Nature of symptoms varied. Almost all of them (N=14) had symptoms in at least two of the following symptom clusters: 1) gastro-intestinal; 2) cardiopulmonary; 3) musculoskeletal/pain and 4) general symptoms (headache, dizziness, memory impairment, concentration difficulties, fatigue). These symptom clusters were identified in a prior study by Fink et al (7) and are also used in the Dutch general practice guideline for MUS (13). A substantial number of patients (N=10) had symptoms in at least three of these symptom clusters. Details on experienced symptoms and other characteristics of the patients are shown in table 1.

Box 1. The PROSPECTS study

The PROSPECTS study is a Dutch longitudinal cohort study following patients (N=325) with persistent physical symptoms (PPS). PPS patients aged between 18-70 years were recruited in general practices (N=218) and in specialized PPS programs of secondary and tertiary care organizations (N=107) across the Netherlands in 2013-2015. Initially patients were followed over a period of three years with five measurements in time (baseline, 6, 12, 24, 36 months of follow-up) (22). In 2017, the follow-up period was extended to a period of five years, adding a 48 and 60 months follow-up measurement. Baseline characteristics and information on the recruitment process and first two years of follow-up have been published elsewhere (19, 23). Over a three years' period, only a minority of the participants (<15%) showed clinical stability in symptom severity and physical functioning.

Definition of PPS: PPS was defined as the presence of physical symptoms, which had lasted at least several weeks and for which no sufficient explanation was found after proper medical examination by a physician. This is in line with the current Dutch multidisciplinary and general practice guidelines for MUS (13, 14).

For peer review only - http://bmjopen.bmj.com/site/about/guidelines.xhtml

Table 1. Patient characteristics

Variable	(n/15)
**Fluctuations/ stability**	
**-Fluctuations in SS and PF**	9/15
**-Stable in SS and PF**	5/15
**-Fluctuations in SS, stable in PF**	1/15
**Symptoms**	
**-Fatigue**	12/15
**-Musculoskeletal pain**	12/15
**-Headache**	6/15
**-Gastro-intestinal symptoms**	5/15
**-Cardiopulmonary symptoms**	3/15
**-Dizziness**	3/15
**Mean age (years, range)**	55.4 years (range 32-73 years)
**Gender**	
**-male**	3/15
**-female**	12/15
**Education**	
**-higher educational level**	4/15
**-intermediate educational level**	4/15
**-lower educational level**	7/15
**Living area**	
**-rural area**	5/15
**-city**	10/15
**Recruitment setting**	
**-general practice**	12/15
**-specialized PPS program**	3/15

SS: symptom severity (based on minimal clinically important differences in PHQ-15)
PF: physical functioning (based on minimal clinically important differences in RAND-36 PCS)

For peer review only

Data collection

Interviews took place between January and April 2019. Based on the preference of the patient, eleven interviews were conducted at the patients' home and four at the research department of the university in a private meeting room. All interviews were digitally recorded. The interviews took 60 minutes on average (range: 33 -93 minutes). Patients received a gift voucher of € 15,-. Participants were told that the main interviewer (HB) is a GP registrar and researcher with an interest in PPS and the fellow interviewer (EW) a medical intern involved in a research project on PPS. Both interviewers are female. HB had received training in qualitative research and was supervised by an experienced qualitative researcher (AdK).

Interviews were loosely structured using a topic guide with relevant areas explored in depth. The main interviewer (HB) emphasized that that all interviews were non-judgmental, confidential and anonymized. She also told the participants the researchers were particularly interested in the course of their symptoms over shorter (days, weeks) and longer (months, years) periods of time. The topic guide consisted of five main topics: (1) the experienced course of symptoms and how symptoms interfered with their daily activities, with special focus on stability and fluctuations over time (day, week, month, year(s)); (2) factors contributing to fluctuations in symptoms; (3) management of symptoms and fluctuations; (4) the role of their social and work environment; (5) the role of the healthcare system and care providers.

Based on our prior quantitative study (19), our preconception was that patients might experience fluctuations in symptoms and that these might be relevant to them. Based on theoretical sampling, we selected 'fluctuating' as well as 'seemingly stable' patients. We expected more prominent accounts on fluctuations in the 'fluctuating' patients. Whilst we had this preconception, we asked open questions in both 'fluctuating' as well as 'seemingly stable' patients about the experienced symptoms over time (a day, a week, a month etc.) when interviewing the patients.

Patients were encouraged to talk freely about their experiences and expand on any aspects they felt were relevant. The topic guide was checked throughout the interview process, no major adjustments were made. All participants received a summary of the interview afterwards for a member check. Fourteen patients responded to the summaries, they confirmed that they recognized their experiences in the summaries and no major changes in content were made.

Data analysis

All interviews were transcribed verbatim and coded using Atlas.ti version 7. The analyzing process was based on thematic analysis according to the six phases described by Braun and Clarke (24). In all phases, at least two researchers were involved (HB, EW, FB) to enrich the analysis. In the first phase, HB, EW and FB familiarized themselves with the data by summarizing and close reading. In the second phase, HB, EW and FB all read and coded the first two interview transcripts, using open coding. Codes were discussed to reach agreement and to improve internal validity. This resulted in an initial code list that was extended when further transcripts were analyzed in pairs following the same strategy. In the following phases, codes were clustered into sub-themes in order to identify patterns in the interviews, after which final themes were identified. HB, EW and FB discussed codes, sub-themes and themes until consensus was reached on all themes. Constant comparison was used in order to understand differences and similarities between patients and within each patient. All results were discussed in the research team to enhance the robustness of the findings. Finally, the report was produced and quotes were extracted that related to the themes. We used the SRQR checklist when writing our report (25).

Results

Three main themes were identified in the analysis: (1) Patterns in symptom fluctuations (2) Perceived causes of symptom exacerbations, and (3) Patients' strategies in gaining control over symptom exacerbations.

For peer review only - http://bmjopen.bmj.com/site/about/guidelines.xhtml

Patterns in symptom fluctuations

All interviewed patients experienced fluctuations in the occurrence and severity of symptoms. This meant that both the selected patients with fluctuations, as well as the seemingly stable patients in our sample experienced fluctuations.

Short-term fluctuations

Fluctuations in symptoms occurred in particular over the day, but also over the week.

"And it varies. One day I am in the shower and I think 'Here it comes'. The next day, well, it can start during the day. And sometimes, very occasionally, I will be fine." (P4, female)

Most patients experienced a gradual worsening of symptoms over the day and work week. Others did not experience a specific pattern. Worsening over the work week was described by all patients who worked.

"If I wake up with little pain, it is a good day. But a day will eventually always end with pain." (P3, female)

"At the end of the week it is usually worse." (P8, male)

Only few patients did not experience a recognizable pattern over the day or week.

Long-term fluctuations

Most patients described exacerbations and remissions of symptoms and how these symptoms influenced their lives over longer periods of time (months-years).

"And I've also had periods when I was able to do other things as well. So there have been periods when things were better, and I could do a little more." (P2, male)

Throughout their lives, a couple of patients described several isolated episodes of symptom exacerbations that lasted at least several months, as well as periods that had been free of symptoms. At the time of the interview, some patients reported a recent increase in symptoms over the weeks before the interview, whereas one patient was free of symptoms at the time of the interview. In some improvement was present, but only for relatively short periods.

"Well, yes, there are bad days and good days, but then there are more bad ones." (P12, female)

In particular for short-term fluctuations patients indicated to continuously search to understand and explain what caused the exacerbations of symptoms, so they could anticipate on and prevent symptoms from worsening.

2. Perceived causes of symptom exacerbations

Overstepping physical limits

Patients described an increase in symptom severity when overstepping their physical limits. Overdoing it was experienced as leading to setbacks with exacerbations of symptoms. Many patients therefore aimed for a certain balance: a balance between their aims and abilities, pushing physical limits but not overdoing it.

"At first I was up and down, all over the place. I really thought 'I'll get over this, I'll do it again, I'll do everything again (…) Well, it takes a couple of years before you really hit the wall and think 'sorry, you can try as hard as you like, you will still have these setbacks.' And then you can start all over again,

For peer review only - http://bmjopen.bmj.com/site/about/guidelines.xhtml

because then you are overstepping your limits." (P11, female)

Some patients described an energy balance. In case of a negative balance, symptoms worsened. Many patients experienced a link between this energy balance and the progression of their symptoms during the day or week.

"You know, it's like 'everybody has an energy span, a range of ability, that is different for every person, and you always want something else'. Only I am usually just confronted with the consequences of this sooner. Because when I think 'I'll keep going now', I'll have a problem tomorrow." (P3, female)

Almost all patients mentioned the importance of respecting their physical limits in order to prevent their symptoms from worsening and to experience fewer fluctuations. Some patients also mentioned the importance of staying active and searching for the right balance, as *not doing enough* also resulted in worse symptoms in these patients.

"It is 'I did either too much, or not enough'. One or the other." (P11, female)

Negative emotions

A couple of patients experienced that their symptoms represented or were exacerbated by negative emotions. One patient linked her symptoms solely to negative emotions and viewed her symptoms as a representation of these emotions. She found the solution in getting a hold of her emotions -that she attributed to her personal situation at that time. By changing her personal situation with the help of her religion, she explained she got rid of these negative feelings. At the time of the interview, she was free of symptoms.

"Well, you know, you are angry, you are sad, or a little depressed. (…) Why is this happening to me? (…) But the physical pains I sometimes had, that was purely because I was sad. You know, that stress that sometimes enters your system. And it also has to do with resignation. How much of your situation do you accept?" (P5, female)

Others mentioned how their symptoms led to worries and negative feelings, and that from their perspective these feelings worsened the symptoms and created a vicious circle.

"At that moment I thought I was dying. And then you get stressed. That's what happens. Then you are in more and more pain. So eventually you get into this vicious circle as a human being. Because when you start thinking 'yes, it is indeed getting worse', that's what happens." (P1, female)

The effect of emotionally stressful events not related to their symptoms was also mentioned as resulting in symptom exacerbations. In these cases patients felt not capable of taking control over their symptoms.

"In an event like that, I won't be able to sit down. There is too much adrenaline in my system. A whole lot of symptoms will follow. Not directly, but after a day or two, when things are calming down a bit." (P3, female)

"We were having a good time together, but then my granddaughter suddenly started to bark at us, she is hitting puberty you know. That hurts. (…) Than you can feel it in your shoulders, you know, because your muscles get more tense." (P12, female)

For some patients it was difficult to acknowledge a relation between negative emotions and their physical symptoms, although they believed there was some connection. One patient with a recent increase in symptoms mentioned the following on this:

For peer review only - http://bmjopen.bmj.com/site/about/guidelines.xhtml

R: Yes, if I'm being honest to myself, I think that it [negative emotions due to job loss] got in my way.
I: Are you experiencing more symptoms since then? Do you connect this?
R: Well, I don't exclude it. (…) When you're honest, I know that myself, you know that it probably plays a role.'' (P8, male)

This view followed after a somatic disease was excluded by the general practitioner and several medical specialist over the last couple of months.

"You can no longer exclude it, when you are physically healthy." (P8, male)

Although a couple of other perceived causes of symptom exacerbations were mentioned (sleep disturbances; focusing on symptoms; food allergies)- these did not have a prominent role in patients' personal accounts and were mentioned as having some impact in addition to the prominent impact of physical limits and/or negative emotions.

3. Patients' strategies in gaining control over symptom exacerbations

Resigning to physical limits

Patients mentioned the importance of respecting their physical limits in order to prevent their symptoms from worsening and to experience fewer ups and downs. They experienced that ignoring their symptoms and not taking their physical limits into account resulted in symptom exacerbations. The lack of recognition and validation of symptoms in the absence of a diagnosis or plausible explanation was mentioned as creating difficulties in respecting and resigning to personal physical limits.

"Only, realistically, I sometimes think 'well, but I don't have a problem'. 'There is nothing wrong with me'. Everything is in working order, so I should be able to just do that. Often this is what gets in the way, like 'it's all in my mind'. You know, why not push through? But then I immediately pay the price." (P3, female)

Although many patients mentioned the importance of resigning to physical limits, they described this ongoing process as challenging and often frustrating. By resigning, we mean that patients expressed the need to take their physical limits seriously and anticipate by limiting their activities in order to prevent exacerbations of symptoms. Resigning to limits was experienced as different from accepting their limits, as many kept struggling with the acceptance of their physical limits. They for example encountered new situations as a result of changing environments and life changes over time, again confronting them with their physical limits.

"I still haven't fully embraced it and am not Zen about it. Because, you know, when I see other mothers. (…) Or when Mum plays tag or something. Then I run ten paces. Can't run too long, or I get myself in trouble. That still frustrates me." (P3, female)

Finding a plausible explanation for the experienced symptoms was seen as helpful in accepting their physical limits. In case an explanation was offered and made sense to the patient, it contributed to the understanding of their symptoms and helped them in acceptance of limitations.

Well, that was really good, because then you finally have an explanation for the symptoms. Because she could also explain where this fatigue comes from. And then, well, you adjust your life to it. So I accepted it, that I can just do less. (P2, male)

For peer review only - http://bmjopen.bmj.com/site/about/guidelines.xhtml

Adjusting daily planning

Most patients eventually adjusted their daily planning and routines to their physical limits and capabilities. They mentioned pacing activities and resting effectively as important strategies in gaining control over symptoms and experiencing fewer ups and downs.

"The entire day I keep in mind what I need and want to do. So if I have a birthday tonight, than I take a nap in the afternoon." (P13, male)

In some patients, incorporating mindfulness and relaxation exercises into their daily routine had positive results regarding their experienced symptoms.

Weighing personal needs and learning to say 'no'

Other patients indicated to have gained control by continuously weighing personal needs. Deciding to participate in joyful activities, while they knew it would exacerbate their symptoms, helped some patients to cope. They described to weigh the personal gain and consequences and in some cases decided to consciously overstep their limits, anticipating an exacerbation of symptoms.

"Yes, and also that you know when you are overdoing it and you still choose to do that, knowing that you will be in serious pain the next day. That makes it easier to accept. The harder you fight, the angrier, I think, you will get and the worse your pain will be." (P11, female)

Whereas in some cases the activity was worth overstepping limits, in other cases patients thoughtfully evaluated the activity as having too little value to them. Learning to say no, in such cases, was also experienced as important in gaining control over symptoms.

"And sometimes I think: I am not going to do that. If I am not well and it's not something I really enjoy. No … I evaluate: is it worthwhile, does it do me any good? Is it something I enjoy? If not, I say no. You also need to learn to say 'No'. I didn't do that when this started." (P12, female)

Discussion

Our findings highlight that fluctuations in symptoms, -and in particular the symptom exacerbations that patients describe over the day and week-, are an important element of symptom experience in patients with PPS. It impacts their daily routines and poses various challenges. Patients attributed the experienced worsening of symptoms in particular to overstepping their physical limits and/ or to the impact of negative emotions. Patients described different strategies in gaining control over symptom exacerbations: by resigning to their physical limits, adjusting their daily planning to their limits and capabilities, weighing personal needs and learning to say 'no'.

As far as we are aware, this is the first qualitative study exploring the experiences with fluctuations in symptoms among patients with PPS. A strength of this study was the fact that patients were recruited in different health care settings throughout the Netherlands and that patients varied with regard to diversity of symptoms and demographic and social characteristics. More female patients were interviewed, but numbers were in line with the balance in the cohort from which we selected the patients (75% female). A limitation of our study, is that all interviewed patients experienced (episodes of) severe PPS for longer periods of time and our findings may be less applicable to patients experiencing mild or symptoms or symptoms of short duration. Another possible limitation is that we had certain preconceptions based on our prior quantitative study and that these might have influenced our findings. We tried to minimize the impact of our preconceptions e.g. by our theoretical sampling

For peer review only - http://bmjopen.bmj.com/site/about/guidelines.xhtml

method in which we included both -'fluctuating' and 'seemingly stable' patients. Although we anticipated differences in experience between these patients, this was in fact not the case.

Our findings correspond to some findings from quantitative studies in CFS. Ecologic momentary assessment established that patients experienced difficulties in balancing their activities in response to symptoms. More fatigue related symptoms and pain predicted more activity limitation whereas feeling subjectively well predicted more all-or-nothing behavior, resulting in ups and downs (15). Pacing activities was helpful in preventing fluctuations in symptoms (16). Comparable quantitative studies in a broader sample of patients with PPS are currently lacking. Our findings, however, suggest that dealing with fluctuations -and in particular anticipating on symptom exacerbations- seems to apply to the broader spectrum of PPS.

In our study, resignation to physical limits was mentioned as a strategy to anticipate on and prevent symptom exacerbations. Having a plausible explanation for symptoms was helpful in acceptance of experienced physical limitations. A prior qualitative study described that in particular patients who displayed acceptance of PPS –as opposed to resignation- shifted their focus towards improving their quality of life (26). Resignation and acceptance seem closely related, but the latter implies to be a later stage in a process of change. Acceptance was also an important condition for symptom improvement (27) and facilitated a process of change towards self-compassion and self-care in patients with PPS (28).

While in our study, resigning to limits was described as an important strategy in anticipating on symptom exacerbations and fluctuations in symptoms, Sowińska and Czachowski (29) described how in their population of Polish patients with PPS (MUS) ignoring symptoms or shifting away attention was reported as one of the most successful ways of coping. These differences are interesting. The Polish patients are likely to represent a different selection of patients with PPS: they were all included in the same general practice and visited psychologists and psychiatrist privately. Cultural differences may play a role as well. Multiple studies (26, 30, 31) highlight PPS patients' concerns that it might be 'all in the mind' and how this often brings shame and the feeling of not having 'a legitimate illness'. In our study, patients also struggled with their physical limits in the absence of a 'legitimate illness'. Although symptom exacerbations were attributed to negative emotions by some patients in our study, patients also indicated initial difficulties in accepting the connection. In a recent study on consultations between GPs and patients, symptoms could be attributed to emotions when patients introduced this link themselves. However, when the GP introduced this link it tended to be denied (32). This again underlines the stigma that still pertains on mental distress and its relation to physical health.

Several of our findings may be helpful in the care for patients with PPS. First, our study again underlines the need to take symptoms and their consequences seriously as a health care provider (HCP), also in the absence of an identifiable disease. Patients with PPS face challenges in dealing with fluctuations in symptoms, and more specific in dealing with symptom exacerbations. Secondly, as a HCP, exploring patients' experiences with symptom exacerbations -with attention paid to the experienced impact of physical limits and negative emotions- might be a useful starting point to gain an understanding of what your patient is struggling with on a daily basis and may create a common ground for supportive care to improve wellbeing and provide illness-based interventions and advice.

Our study highlights that fluctuations in symptoms are an important element of the experienced symptoms. More longitudinal research into short-term fluctuations in experienced symptoms in a broad sample of patients with PPS, for example by the experience sampling method (ESM), could provide useful new insights. ESM can reveal how symptom experience relates to implicit patterns of thought, experience and behavior (33). Another valuable area of research could be the different strategies of gaining control over symptom exacerbations and their impact on functional health and wellbeing.

For peer review only - http://bmjopen.bmj.com/site/about/guidelines.xhtml

Acknowledgements

We want to thank all the patients that took part in this study for sharing their experiences.

Funding

This work was supported by grants of ZonMw [funding number 839110018] and the Stoffels-Hornstra Foundation. The funding sources had no role in the design, analyses and interpretation of this study.

Author Statement

HB, NCvD, JCvdW, JD and HvdH developed the study protocol. AdK and ToH provided feedback on the study protocol. HB and EW contributed to the development of the topic guide, collected and analysed the data and interpreted the results. FB analysed the data and interpreted the results. AdK provided feedback on the data collection and analysis. HB drafted the manuscript. All authors read, provided critical revisions and approved the manuscript.

Competing interests

Apart from the institutional grants mentioned above, there are no other potential competing interests to report.

Data sharing statement

No additional data are available.

References

1. Picariello F, Ali S, Moss-Morris R, Chalder T. The most popular terms for medically unexplained symptoms: the views of CFS patients. J Psychosom Res. 2015;78(5):420-6.
2. Marks EM, Hunter MS. Medically Unexplained Symptoms: an acceptable term? Br J Pain. 2015;9(2):109-14.
3. Konnopka A, Schaefert R, Heinrich S, Kaufmann C, Luppa M, Herzog W, et al. Economics of medically unexplained symptoms: a systematic review of the literature. Psychother Psychosom. 2012;81(5):265-75.
4. Rask MT, Ornbol E, Rosendal M, Fink P. Long-term outcome of bodily distress syndrome in primary care: A follow-up study on health care costs, work disability, and self-rated health. Psychosom Med. 2017;79(3):345-57.
5. Nimnuan C, Hotopf M, Wessely S. Medically unexplained symptoms: an epidemiological study in seven specialities. J Psychosom Res. 2001;51(1):361-7.
6. Fink P, Schroder A. One single diagnosis, bodily distress syndrome, succeeded to capture 10 diagnostic categories of functional somatic syndromes and somatoform disorders. J Psychosom Res. 2010;68(5):415-26.
7. Fink P, Toft T, Hansen MS, Ornbol E, Olesen F. Symptoms and syndromes of bodily distress: an exploratory study of 978 internal medical, neurological, and primary care patients. Psychosom Med. 2007;69(1):30-9.
8. Budtz-Lilly A, Schroder A, Rask MT, Fink P, Vestergaard M, Rosendal M. Bodily distress syndrome: A new diagnosis for functional disorders in primary care? BMC Fam Pract. 2015;16:180.
9. Wessely S, White PD. There is only one functional somatic syndrome. Br J Psychiatry. 2004;185:95-6.
10. Jones B. Why we should stop talking about 'medically unexplained symptoms' etc. - the problem of overgeneralisation for research and treatment. J Psychosom Res. 2019;127:109835.

For peer review only - http://bmjopen.bmj.com/site/about/guidelines.xhtml

11. Abbi B, Natelson BH. Is chronic fatigue syndrome the same illness as fibromyalgia: evaluating the 'single syndrome' hypothesis. QJM. 2013;106(1):3-9.
12. White PD. Chronic fatigue syndrome: Is it one discrete syndrome or many? Implications for the "one vs. many" functional somatic syndromes debate. J Psychosom Res. 2010;68(5):455-9.
13. Olde Hartman T, Blankenstein AH, Molenaar B, Bentz van den Berg B, van der Horst HE, Arnold I, et al. [Dutch College of GPs' guideline on medically unexplained physical symptoms] (In Dutch). Huisarts Wet. 2013;56(5):222-30.
14. van der Feltz-Cornelis CM, Swinkels JA, Blankenstein AH, Hoedeman R, Keuter EJ. The Dutch multidisciplinary guideline entitled 'Medically unexplained physical symptoms and somatoform disorder' (In Dutch). Ned Tijdschr Geneeskd. 2011;155(18):A1244..
15. Band R, Barrowclough C, Caldwell K, Emsley R, Wearden A. Activity patterns in response to symptoms in patients being treated for chronic fatigue syndrome: An experience sampling methodology study. Health Psychol. 2017;36(3):264-9.
16. Meeus M, van Eupen I, van Baarle E, De Boeck V, Luyckx A, Kos D, et al. Symptom fluctuations and daily physical activity in patients with chronic fatigue syndrome: a case-control study. Arch Phys Med Rehabil. 2011;92(11):1820-6.
17. Zautra AJ, Fasman R, Parish BP, Davis MC. Daily fatigue in women with osteoarthritis, rheumatoid arthritis, and fibromyalgia. Pain. 2007;128(1-2):128-35.
18. Olde Hartman TC, Borghuis MS, Lucassen PL, van de Laar FA, Speckens AE, van Weel C. Medically unexplained symptoms, somatisation disorder and hypochondriasis: course and prognosis. A systematic review. J Psychosom Res. 2009;66(5):363-77.
19. Claassen-van Dessel N, van der Wouden JC, Hoekstra T, Dekker J, van der Horst HE. The 2-year course of Medically Unexplained Physical Symptoms (MUPS) in terms of symptom severity and functional status: results of the PROSPECTS cohort study. J Psychosom Res. 2018;104:76-87.
20. Kroenke K, Spitzer RL, Williams JB. The PHQ-15: validity of a new measure for evaluating the severity of somatic symptoms. Psychosom Med. 2002;64(2):258-66.
21. VanderZee KI, Sanderman R, Heyink JW, de Haes H. Psychometric qualities of the RAND 36-Item Health Survey 1.0: a multidimensional measure of general health status. Int J Behav Med. 1996;3(2):104-22.
22. van Dessel N, Leone SS, van der Wouden JC, Dekker J, van der Horst HE. The PROSPECTS study: design of a prospective cohort study on prognosis and perpetuating factors of medically unexplained physical symptoms (MUPS). J Psychosom Res. 2014; 76: 200-206.
23. Claassen- van Dessel N, van der Wouden JC, Dekker J, van der Horst HE. Clinical value of DSM IV and DSM 5 criteria for diagnosing the most prevalent somatoform disorders in patients with medically unexplained physical symptoms (MUPS). J Psychosom Res. 2016; 82: 4-10.
24. Braun V and Clarke V. Using thematic analysis in psychology. Qual Res Psychol. 2006(3):77-101.
25. O'Brien BC, Harris IB, Beckman TJ, Reed DA, Cook DA. Standards for reporting qualitative research: a synthesis of recommendations. Acad Med. 2014;89(9):1245-51.
26. Kornelsen J, Atkins C, Brownell K, Woollard R. The meaning of patient experiences of medically unexplained physical symptoms. Qual Health Res. 2016;26(3):367-76.
27. Claassen-van Dessel N Velzeboer FR, van der Wouden JC, den Boer C, Dekker J, van der Horst HE. Patients' perspectives on improvement of medically unexplained physical symptoms: a qualitative analysis. Ment Health Fam Med. 2015;11(02).
28. van Ravesteijn HJ, Suijkerbuijk YB, Langbroek JA, Muskens E, Lucassen PL, van Weel C, et al. Mindfulness-based cognitive therapy (MBCT) for patients with medically unexplained symptoms: process of change. J Psychosom Res. 2014;77(1):27-33.
29. Sowinska A, Czachowski S. Patients' experiences of living with medically unexplained symptoms (MUS): a qualitative study. BMC Fam Pract. 2018;19(1):23.
30. Stone L. Blame, shame and hopelessness: medically unexplained symptoms and the 'heartsink' experience. Aust Fam Physician. 2014;43(4):191-5.

For peer review only - http://bmjopen.bmj.com/site/about/guidelines.xhtml

31. Nettleton S, Watt I, O'Malley L, Duffey P. Understanding the narratives of people who live with medically unexplained illness. Patient education and counseling. 2005;56(2):205-10.
32. Bekhuis E, Gol J, Burton C, Rosmalen J. Patients' descriptions of the relation between physical symptoms and negative emotions: a qualitative analysis of primary care consultations. Br J Gen Pract. 2020;70(691):e78-e85.
33. van Os J, Verhagen S, Marsman A, Peeters F, Bak M, Marcelis M, et al. The experience sampling method as an mHealth tool to support self-monitoring, self-insight, and personalized health care in clinical practice. Depress Anxiety. 2017;34(6):481-93.

For peer review only

For peer review only - http://bmjopen.bmj.com/site/about/guidelines.xhtml

Reporting checklist for qualitative study.

Based on the SRQR guidelines.

Instructions to authors

Complete this checklist by entering the page numbers from your manuscript where readers will find each of the items listed below.

Your article may not currently address all the items on the checklist. Please modify your text to include the missing information. If you are certain that an item does not apply, please write "n/a" and provide a short explanation.

Upload your completed checklist as an extra file when you submit to a journal.

In your methods section, say that you used the SRQRreporting guidelines, and cite them as:

O'Brien BC, Harris IB, Beckman TJ, Reed DA, Cook DA. Standards for reporting qualitative research: a synthesis of recommendations. Acad Med. 2014;89(9):1245-1251.

		Reporting Item	Page Number
**Title**			
	#1	Concise description of the nature and topic of the study identifying the study as qualitative or indicating the approach (e.g. ethnography, grounded theory) or data collection methods (e.g. interview, focus group) is recommended	1
**Abstract**			
	#2	Summary of the key elements of the study using the abstract format of the intended publication; typically includes background, purpose, methods, results and conclusions	2
**Introduction**			
Problem formulation	#3	Description and significance of the problem / phenomenon studied: review of relevant theory and empirical work; problem statement	4

For peer review only

Purpose or research question	#4	Purpose of the study and specific objectives or questions	4
**Methods**			
Qualitative approach and research paradigm	#5	Qualitative approach (e.g. ethnography, grounded theory, case study, phenomenolgy, narrative research) and guiding theory if appropriate; identifying the research paradigm (e.g. postpositivist, constructivist / interpretivist) is also recommended; rationale. The rationale should briefly discuss the justification for choosing that theory, approach, method or technique rather than other options available; the assumptions and limitations implicit in those choices and how those choices influence study conclusions and transferability. As appropriate the rationale for several items might be discussed together.	4-7
Researcher characteristics and reflexivity	#6	Researchers' characteristics that may influence the research, including personal attributes, qualifications / experience, relationship with participants, assumptions and / or presuppositions; potential or actual interaction between researchers' characteristics and the research questions, approach, methods, results and / or transferability	7
Context	#7	Setting / site and salient contextual factors; rationale	4,6,7
Sampling strategy	#8	How and why research participants, documents, or events were selected; criteria for deciding when no further sampling was necessary (e.g. sampling saturation); rationale	4,5
Ethical issues pertaining to human subjects	#9	Documentation of approval by an appropriate ethics review board and participant consent, or explanation for lack thereof; other confidentiality and data security issues	4
Data collection methods	#10	Types of data collected; details of data collection procedures including (as appropriate) start and stop dates of data collection and analysis, iterative	7

For peer review only

		process, triangulation of sources / methods, and modification of procedures in response to evolving study findings; rationale	
Data collection instruments and technologies	#11	Description of instruments (e.g. interview guides, questionnaires) and devices (e.g. audio recorders) used for data collection; if / how the instruments(s) changed over the course of the study	7
Units of study	#12	Number and relevant characteristics of participants, documents, or events included in the study; level of participation (could be reported in results)	5,6
Data processing	#13	Methods for processing data prior to and during analysis, including transcription, data entry, data management and security, verification of data integrity, data coding, and anonymisation / deidentification of excerpts	7
Data analysis	#14	Process by which inferences, themes, etc. were identified and developed, including the researchers involved in data analysis; usually references a specific paradigm or approach; rationale	7
Techniques to enhance trustworthiness	#15	Techniques to enhance trustworthiness and credibility of data analysis (e.g. member checking, audit trail, triangulation); rationale	7
**Results/findings**			
Syntheses and interpretation	#16	Main findings (e.g. interpretations, inferences, and themes); might include development of a theory or model, or integration with prior research or theory	8,9,10,11
Links to empirical data	#17	Evidence (e.g. quotes, field notes, text excerpts, photographs) to substantiate analytic findings	8,9,10,11
**Discussion**			
Intergration with prior work, implications, transferability and contribution(s) to the field	#18	Short summary of main findings; explanation of how findings and conclusions connect to, support, elaborate on, or challenge conclusions of earlier scholarship; discussion of scope of application /	11,12

For peer review only

		generalizability; identification of unique contributions(s) to scholarship in a discipline or field	
Limitations	#19	Trustworthiness and limitations of findings	11,12
**Other**			
Conflicts of interest	#20	Potential sources of influence of perceived influence on study conduct and conclusions; how these were managed	11
Funding	#21	Sources of funding and other support; role of funders in data collection, interpretation and reporting	13

The SRQR checklist is distributed with permission of Wolters Kluwer © 2014 by the Association of American Medical Colleges. This checklist was completed on 15. November 2019 using https://www.goodreports.org/, a tool made by the EQUATOR Network in collaboration with Penelope.ai

For peer review only

BMJ Open

Patients' experiences with fluctuations in persistent physical symptoms: a qualitative study

Journal:	*BMJ Open*
Manuscript ID	bmjopen-2019-035833.R2
Article Type:	Original research
Date Submitted by the Author:	02-May-2020
Complete List of Authors:	Barends, Hieke; Amsterdam UMC - VUMC location, General Practice and Elderly Care Medicine; Amsterdam Public Health research insitute<br>Walstock, Ella; Amsterdam UMC - VUMC location, Department of General Practice and Elderly Care Medicine; Amsterdam Public Health research institute<br>Botman, Femke; Amsterdam UMC - VUMC location, Department of General Practice and Elderly Care Medicine; Amsterdam Public Health research institute<br>de Kruif , Anja ; Vrije Universiteit Amsterdam, Department of Health Sciences, Faculty of Science<br>Claassen, Nikki; Amsterdam UMC - VUMC location, Department of General Practice and Elderly Care Medicine; Amsterdam Public Health research institute<br>van der Wouden, Johannes; Amsterdam UMC - VUMC location, Department of General Practice and Elderly Care Medicine; Amsterdam Public Health research institute<br>Olde Hartman, Tim; Radboud University Nijmegen Medical Center, Department of Primary and Community Care; Radboud University Donders Institute for Brain Cognition and Behaviour<br>Dekker, Joost; Amsterdam UMC - VUMC location, Department of Rehabilitation Medicine and Department of Psychiatry; Amsterdam Public Health research institute<br>van der Horst, Henriette; Amsterdam UMC - VUMC location, Department of General Practice and Elderly Care Medicine; Amsterdam Public Health research institute
**Primary Subject Heading**:	Qualitative research
Secondary Subject Heading:	General practice / Family practice, Patient-centred medicine
Keywords:	QUALITATIVE RESEARCH, PRIMARY CARE, GENERAL MEDICINE (see Internal Medicine)

SCHOLARONE™ Manuscripts

For peer review only - http://bmjopen.bmj.com/site/about/guidelines.xhtml

I, the Submitting Author has the right to grant and does grant on behalf of all authors of the Work (as defined in the below author licence), an exclusive licence and/or a non-exclusive licence for contributions from authors who are: i) UK Crown employees; ii) where BMJ has agreed a CC-BY licence shall apply, and/or iii) in accordance with the terms applicable for US Federal Government officers or employees acting as part of their official duties; on a worldwide, perpetual, irrevocable, royalty-free basis to BMJ Publishing Group Ltd ("BMJ") its licensees and where the relevant Journal is co-owned by BMJ to the co-owners of the Journal, to publish the Work in this journal and any other BMJ products and to exploit all rights, as set out in our licence.

The Submitting Author accepts and understands that any supply made under these terms is made by BMJ to the Submitting Author unless you are acting as an employee on behalf of your employer or a postgraduate student of an affiliated institution which is paying any applicable article publishing charge ("APC") for Open Access articles. Where the Submitting Author wishes to make the Work available on an Open Access basis (and intends to pay the relevant APC), the terms of reuse of such Open Access shall be governed by a Creative Commons licence – details of these licences and which Creative Commons licence will apply to this Work are set out in our licence referred to above.

Other than as permitted in any relevant BMJ Author's Self Archiving Policies, I confirm this Work has not been accepted for publication elsewhere, is not being considered for publication elsewhere and does not duplicate material already published. I confirm all authors consent to publication of this Work and authorise the granting of this licence.

For peer review only

Patients' experiences with fluctuations in persistent physical symptoms: a qualitative study

Hieke Barends, MD[1,2]*
Ella Walstock, MSc, medical student[1,2]
Femke Botman, MSc, medical anthropologist[1,2]
Anja (JThCM) de Kruif, MSc, health scientist, senior researcher[3]
Nikki Claassen, MD, PhD, general practitioner[1,2]
Johannes C. van der Wouden, PhD, sociologist and associate professor[1,2]
Tim olde Hartman, MD, PhD, general practitioner, senior researcher[5]
Joost Dekker, PhD, psychologist, professor of Allied Health Care [2,4]
Henriëtte E. van der Horst, MD, PhD, general practitioner, professor of General Practice[1,2]

[1] Amsterdam UMC, Vrije Universiteit Amsterdam, Department of General Practice and Elderly Care Medicine, the Netherlands
[2] Amsterdam Public Health research institute
[3] Department of Health Sciences, Faculty of Science, Amsterdam Public Health Institute, Vrije Universiteit Amsterdam, the Netherlands
[4] Amsterdam UMC, Vrije Universiteit Amsterdam, Department of Rehabilitation Medicine and Department of Psychiatry, the Netherlands
[5] Department of Primary and Community Care, Donders Institute for Brain, Cognition and Behaviour, Radboud University Nijmegen Medical Center, Nijmegen, the Netherlands

Corresponding author:
Hieke Barends
Amsterdam UMC, Vrije Universiteit Amsterdam
Department of General Practice and Elderly Care Medicine
Amsterdam Public Health research institute,
Medical Faculty, room C-378
Van der Boechorststraat 7, 1081 BT Amsterdam
Tel: +31 20-4449678
Email: h.barends@amsterdamumc.nl

Word count: 4269 words

Email of all other authors: ellawalstock@gmail.com; f.botman@amsterdamumc.nl; anja.de.kruif@vu.nl; nclaassen.vandessel@gmail.com; j.vanderwouden@amsterdamumc.nl; Tim.OldeHartman@radboudumc.nl; j.dekker@amsterdamumc.nl; he.vanderhorst@amsterdamumc.nl

For peer review only - http://bmjopen.bmj.com/site/about/guidelines.xhtml

Abstract

Objectives: To explore patients' experiences with fluctuations in persistent physical symptoms (PPS) and to understand which factors -from their viewpoint- play a role in these fluctuations.

Design: Qualitative study using semi-structured interviews and thematic content analysis.

Setting: This qualitative study is part of a multi-center prospective cohort study on the course of PPS. Patients were recruited in general practices and specialized treatment facilities for PPS throughout the Netherlands.

Participants: Interviews were conducted with a sample of fifteen patients with PPS to explore their experiences with fluctuations in symptom severity.

Results: We identified three themes in the analysis: (1) Patterns in symptom fluctuations (2) Perceived causes of symptom exacerbations, and (3) Patients' strategies in gaining control over symptom exacerbations. Daily and weekly fluctuations in symptoms were an important element in patients' experiences. In particular anticipating on the worsening of symptoms impacted their daily routines and posed various challenges. Symptom exacerbations were attributed to overstepping physical limits and/or the impact of negative emotions. Resigning to physical limits, adjusting ones daily planning, weighing personal needs and learning to say 'no' were described as different strategies in gaining control over symptom exacerbations.

Conclusions: Fluctuations in the severity of symptoms -and in particular daily and weekly symptom exacerbations- are an important element of the symptom experience in patients with PPS and poses various challenges. Patients attributed symptom exacerbation to overstepping physical limits and/or negative emotions. Patients described different strategies in gaining control over symptom exacerbations.

Keywords

Patients experiences
Fluctuations in symptoms
Qualitative study
Persistent Physical Symptoms
Medically Unexplained Symptoms

For peer review only

Article Summary

Strengths and limitations of this study

- Qualitative research was applied to understand patients' experiences with fluctuations of symptoms and factors playing a role in these fluctuations from their perspective.

- Our study highlights that fluctuations in the experienced severity of symptoms -and in particular daily and weekly symptom exacerbations- are an important element of the symptom experience in PPS and deserve more attention in care for these patients and in research.

- Patients were recruited in general practices as well as in specialized PPS programs in different parts of the Netherlands, and in that regard represent a broad sample of patients with persistent physical symptoms.

- All of the recruited patients experienced (episodes of) severe PPS and most experienced symptoms for an extensive period of time (>5 years), therefore our findings may be less applicable to patients experiencing mild or moderate symptoms or symptoms of short duration.

For peer review only

Introduction

Patients with physical symptoms not attributable to verifiable, conventionally defined diseases are common in all medical settings. These symptoms are often referred to as "medically unexplained symptoms" (MUS). A recent and perhaps more appropriate term -putting less emphasis on the mind-body dualism in the origin of symptoms- is persistent physical symptoms (PPS) (1, 2). When these symptoms persist, they can have a severe impact on patients' quality of life and functional capabilities and also on society due to high medical care utilization and loss of productivity (3, 4).

There has been extensive debate about definitions and terminology in this field of research. Whereas some emphasize commonalities and overlap in symptoms and characteristics (5-8), others differentiate between particular functional somatic syndromes (FSS), such as fibromyalgia, chronic fatigue syndrome and irritable bowel syndrome (9-11). The importance of studying both similarities as well as differences has also been highlighted (12). In this study we focus on similarities and overlap in patients' symptom experiences. We defined PPS as symptoms, which last at least several weeks and for which no sufficient somatic explanation is found after proper medical examination by a physician. This is in line with the current Dutch multidisciplinary and general practice guidelines for MUS (PPS) (13, 14). So, by definition our umbrella term PPS may also cover several FSS.

Fluctuations in symptoms have been described in several quantitative studies in patients with FFS (15-17). Most studies on the course of PPS in a broad sample of patients used a single follow-up measurement in time to determine improvement or deterioration. According to a number of studies conducted in primary and secondary health care settings, 50-75% of patients with PPS showed symptom improvement over time, whereas 10-30% worsened (18). In a cohort study that we conducted on the course of PPS we found improvement (63%) and deterioration (27%) rates that were in line with prior literature, when using total changes scores based on two measurements. However, when four available measurements were taken into account, the temporal stability of these outcomes was limited, as intra-patient fluctuations were highly prevalent (19). These findings suggest that most patients with PPS might experience exacerbations and remissions in symptoms.

To the best of our knowledge, no prior qualitative study focused specifically on fluctuations of symptoms in PPS. Understanding the experiences of fluctuations in symptom severity may help medical professionals in providing care for these patients. This knowledge may enable them to understand what their patients are dealing with and to provide better guidance and support to patients with PPS. Therefore, the aims of this qualitative study were to explore patients' experiences with fluctuations in the severity of symptoms and -if present- to gain insight into factors influencing fluctuations in their symptoms from the patients' perspective.

Methods

Study design

The present study was part of a larger prospective cohort study that monitors the course of symptoms and physical functioning in patients with PPS. We chose a qualitative design and conducted semi-structured (in-depth) interviews, to obtain information about the experiences of patients with PPS. The institutional review board of the Amsterdam UMC (IRB00002991) approved the research protocol (No. 2018.483).

Patient and public involvement

Patients or the public were not actively involved in the design, conduct, reporting or dissemination plans of our research.

For peer review only - http://bmjopen.bmj.com/site/about/guidelines.xhtml

For peer review only

Participants

Participants were selected from the PROSPECTS study (see Box 1). For the PROSPECTS study, patients filled in questionnaires about the nature and severity of their symptoms (Patient Health Questionnaire-15 (PHQ-15), 0-30 scale (20)) and physical functioning (RAND-36 Physical Component Summary (PCS), 0-100 scale (21)) among other questionnaires. We wanted to include patients with fluctuations as well as patients with a (seemingly) stable course of their PPS, because symptom experiences in terms of stability and fluctuations might differ between these patients. Therefore, we selected patients who: (1) showed either clinically relevant fluctuations or clinical stability (based on minimal clinically important differences) in symptom severity (PHQ-15) and physical functioning (RAND-36 PCS) over a three-year time period and (2) had given informed consent to be contacted for future research. We used purposive sampling to ensure a diversity of participants in terms of nature of symptoms, age, gender, social characteristics (educational level, living in a rural/ urban area) and recruitment setting. Over a three years' period, only a minority of the participants (<15%) showed clinical stability in symptom severity and physical functioning.

Patients were approached by phone by HB or EW. In total, 21 patients were contacted. Two patients were not willing to participate because of personal reasons, three patients refused because of time constraints. One patient cancelled the interview appointment due to work-related reasons. All selected patients provided written informed consent.

Fifteen patients agreed to participate. All of the recruited patients experienced (episodes of) severe PPS and most experienced symptoms for an extensive period of time (>5 years). Nature of symptoms varied. Almost all of them (N=14) had symptoms in at least two of the following symptom clusters: 1) gastro-intestinal; 2) cardiopulmonary; 3) musculoskeletal/pain and 4) general symptoms (headache, dizziness, memory impairment, concentration difficulties, fatigue). These symptom clusters were identified in a prior study by Fink et al (7) and are also used in the Dutch general practice guideline for MUS (13). A substantial number of patients (N=10) had symptoms in at least three of these symptom clusters. Details on experienced symptoms and other characteristics of the patients are shown in table 1.

Box 1. The PROSPECTS study

The PROSPECTS study is a Dutch longitudinal cohort study following patients (N=325) with persistent physical symptoms (PPS). PPS patients aged between 18-70 years were recruited in general practices (N=218) and in specialized PPS programs of secondary and tertiary care organizations (N=107) across the Netherlands in 2013-2015. Initially patients were followed over a period of three years with five measurements in time (baseline, 6, 12, 24, 36 months of follow-up) (22). In 2017, the follow-up period was extended to a period of five years, adding a 48 and 60 months follow-up measurement. Baseline characteristics and information on the recruitment process and first two years of follow-up have been published elsewhere (19, 23). Over a three years' period, only a minority of the participants (<15%) showed clinical stability in symptom severity and physical functioning.

<u>Definition of PPS</u>: PPS was defined as the presence of physical symptoms, which had lasted at least several weeks and for which no sufficient explanation was found after proper medical examination by a physician. This is in line with the current Dutch multidisciplinary and general practice guidelines for MUS (13, 14).

For peer review only - http://bmjopen.bmj.com/site/about/guidelines.xhtml

Table 1. Patient characteristics

Variable	(n/15)
**Fluctuations/ stability**	
-Fluctuations in SS and PF	9/15
-Stable in SS and PF	5/15
-Fluctuations in SS, stable in PF	1/15
**Symptoms**	
-Fatigue	12/15
-Musculoskeletal pain	12/15
-Headache	6/15
-Gastro-intestinal symptoms	5/15
-Cardiopulmonary symptoms	3/15
-Dizziness	3/15
**Mean age (years, range)**	55.4 years (range 32-73 years)
**Gender**	
-male	3/15
-female	12/15
**Education**	
-higher educational level	4/15
-intermediate educational level	4/15
-lower educational level	7/15
**Living area**	
-rural area	5/15
-city	10/15
**Recruitment setting**	
-general practice	12/15
-specialized PPS program	3/15

SS: symptom severity (based on minimal clinically important differences in PHQ-15)
PF: physical functioning (based on minimal clinically important differences in RAND-36 PCS)

For peer review only - http://bmjopen.bmj.com/site/about/guidelines.xhtml

Data collection

Interviews took place between January and April 2019. Based on the preference of the patient, eleven interviews were conducted at the patients' home and four at the research department of the university in a private meeting room. All interviews were digitally recorded. The interviews took 60 minutes on average (range: 33 -93 minutes). Patients received a gift voucher of € 15,-. Participants were told that the main interviewer (HB) is a GP registrar and researcher with an interest in PPS and the fellow interviewer (EW) a medical intern involved in a research project on PPS. Both interviewers are female. HB had received training in qualitative research and was supervised by an experienced qualitative researcher (AdK).

Interviews were loosely structured using a topic guide with relevant areas explored in depth. The main interviewer (HB) emphasized that that all interviews were non-judgmental, confidential and anonymized. She also told the participants the researchers were particularly interested in the course of their symptoms over shorter (days, weeks) and longer (months, years) periods of time. The topic guide consisted of five main topics: (1) the experienced course of symptoms and how symptoms interfered with their daily activities, with special focus on stability and fluctuations over time (day, week, month, year(s)); (2) factors contributing to fluctuations in symptoms; (3) management of symptoms and fluctuations; (4) the role of their social and work environment; (5) the role of the healthcare system and care providers.

Based on our prior quantitative study (19), our preconception was that patients might experience fluctuations in symptoms and that these might be relevant to them. Based on theoretical sampling, we selected 'fluctuating' as well as 'seemingly stable' patients. We expected more prominent accounts on fluctuations in the 'fluctuating' patients. Whilst we had this preconception, we asked open questions in both 'fluctuating' as well as 'seemingly stable' patients about the experienced symptoms over time (a day, a week, a month etc.) when interviewing the patients.

Patients were encouraged to talk freely about their experiences and expand on any aspects they felt were relevant. The topic guide was checked throughout the interview process, no major adjustments were made. All participants received a summary of the interview afterwards for a member check. Fourteen patients responded to the summaries, they confirmed that they recognized their experiences in the summaries and no major changes in content were made.

Data analysis

All interviews were transcribed verbatim and coded using Atlas.ti version 7. The analyzing process was based on thematic analysis according to the six phases described by Braun and Clarke (24). In all phases, at least two researchers were involved (HB, EW, FB) to enrich the analysis. In the first phase, HB, EW and FB familiarized themselves with the data by summarizing and close reading. In the second phase, HB, EW and FB all read and coded the first two interview transcripts, using open coding. Codes were discussed to reach agreement and to improve internal validity. This resulted in an initial code list that was extended when further transcripts were analyzed in pairs following the same strategy. In the following phases, codes were clustered into sub-themes in order to identify patterns in the interviews, after which final themes were identified. HB, EW and FB discussed codes, sub-themes and themes until consensus was reached on all themes. Constant comparison was used in order to understand differences and similarities between patients and within each patient. All results were discussed in the research team to enhance the robustness of the findings. Finally, the report was produced and quotes were extracted that related to the themes. We used the SRQR checklist when writing our report (25).

Results

Three main themes were identified in the analysis: (1) Patterns in symptom fluctuations (2) Perceived causes of symptom exacerbations, and (3) Patients' strategies in gaining control over symptom exacerbations.

For peer review only - http://bmjopen.bmj.com/site/about/guidelines.xhtml

Patterns in symptom fluctuations

All interviewed patients experienced fluctuations in the occurrence and severity of symptoms. This meant that both the selected patients with fluctuations, as well as the seemingly stable patients in our sample experienced fluctuations.

Short-term fluctuations

Fluctuations in symptoms occurred in particular over the day, but also over the week.

"And it varies. One day I am in the shower and I think 'Here it comes'. The next day, well, it can start during the day. And sometimes, very occasionally, I will be fine." (P4, female)

Most patients experienced a gradual worsening of symptoms over the day and work week. Others did not experience a specific pattern. Worsening over the work week was described by all patients who worked.

"If I wake up with little pain, it is a good day. But a day will eventually always end with pain." (P3, female)

"At the end of the week it is usually worse." (P8, male)

Only few patients did not experience a recognizable pattern over the day or week.

Long-term fluctuations

Most patients described exacerbations and remissions of symptoms and how these symptoms influenced their lives over longer periods of time (months-years).

"And I've also had periods when I was able to do other things as well. So there have been periods when things were better, and I could do a little more." (P2, male)

Throughout their lives, a couple of patients described several isolated episodes of symptom exacerbations that lasted at least several months, as well as periods that had been free of symptoms. At the time of the interview, some patients reported a recent increase in symptoms over the weeks before the interview, whereas one patient was free of symptoms at the time of the interview. In some improvement was present, but only for relatively short periods.

"Well, yes, there are bad days and good days, but then there are more bad ones." (P12, female)

In particular for short-term fluctuations patients indicated to continuously search to understand and explain what caused the exacerbations of symptoms, so they could anticipate on and prevent symptoms from worsening.

2. Perceived causes of symptom exacerbations

Overstepping physical limits

Patients described an increase in symptom severity when overstepping their physical limits. Overdoing it was experienced as leading to setbacks with exacerbations of symptoms. Many patients therefore aimed for a certain balance: a balance between their aims and abilities, pushing physical limits but not overdoing it.

"At first I was up and down, all over the place. I really thought 'I'll get over this, I'll do it again, I'll do everything again (…) Well, it takes a couple of years before you really hit the wall and think 'sorry, you can try as hard as you like, you will still have these setbacks.' And then you can start all over again,

For peer review only - http://bmjopen.bmj.com/site/about/guidelines.xhtml

because then you are overstepping your limits." (P11, female)

Some patients described an energy balance. In case of a negative balance, symptoms worsened. Many patients experienced a link between this energy balance and the progression of their symptoms during the day or week.

"You know, it's like 'everybody has an energy span, a range of ability, that is different for every person, and you always want something else'. Only I am usually just confronted with the consequences of this sooner. Because when I think 'I'll keep going now', I'll have a problem tomorrow." (P3, female)

Almost all patients mentioned the importance of respecting their physical limits in order to prevent their symptoms from worsening and to experience fewer fluctuations. Some patients also mentioned the importance of staying active and searching for the right balance, as *not doing enough* also resulted in worse symptoms in these patients.

"It is 'I did either too much, or not enough'. One or the other." (P11, female)

Negative emotions

A couple of patients experienced that their symptoms represented or were exacerbated by negative emotions. One patient linked her symptoms solely to negative emotions and viewed her symptoms as a representation of these emotions. She found the solution in getting a hold of her emotions -that she attributed to her personal situation at that time. By changing her personal situation with the help of her religion, she explained she got rid of these negative feelings. At the time of the interview, she was free of symptoms.

"Well, you know, you are angry, you are sad, or a little depressed. (…) Why is this happening to me? (…) But the physical pains I sometimes had, that was purely because I was sad. You know, that stress that sometimes enters your system. And it also has to do with resignation. How much of your situation do you accept?" (P5, female)

Others mentioned how their symptoms led to worries and negative feelings, and that from their perspective these feelings worsened the symptoms and created a vicious circle.

"At that moment I thought I was dying. And then you get stressed. That's what happens. Then you are in more and more pain. So eventually you get into this vicious circle as a human being. Because when you start thinking 'yes, it is indeed getting worse', that's what happens." (P1, female)

The effect of emotionally stressful events not related to their symptoms was also mentioned as resulting in symptom exacerbations. In these cases patients felt not capable of taking control over their symptoms.

"In an event like that, I won't be able to sit down. There is too much adrenaline in my system. A whole lot of symptoms will follow. Not directly, but after a day or two, when things are calming down a bit." (P3, female)

"We were having a good time together, but then my granddaughter suddenly started to bark at us, she is hitting puberty you know. That hurts. (…) Than you can feel it in your shoulders, you know, because your muscles get more tense." (P12, female)

For some patients it was difficult to acknowledge a relation between negative emotions and their physical symptoms, although they believed there was some connection. One patient with a recent increase in symptoms mentioned the following on this:

For peer review only - http://bmjopen.bmj.com/site/about/guidelines.xhtml

R: Yes, if I'm being honest to myself, I think that it [negative emotions due to job loss] got in my way.
I: Are you experiencing more symptoms since then? Do you connect this?
R: Well, I don't exclude it. (…) When you're honest, I know that myself, you know that it probably plays a role.'' (P8, male)

This view followed after a somatic disease was excluded by the general practitioner and several medical specialist over the last couple of months.

"You can no longer exclude it, when you are physically healthy." (P8, male)

Although a couple of other perceived causes of symptom exacerbations were mentioned (sleep disturbances; focusing on symptoms; food allergies)- these did not have a prominent role in patients' personal accounts and were mentioned as having some impact in addition to the prominent impact of physical limits and/or negative emotions.

3. Patients' strategies in gaining control over symptom exacerbations

Resigning to physical limits

Patients mentioned the importance of respecting their physical limits in order to prevent their symptoms from worsening and to experience fewer ups and downs. They experienced that ignoring their symptoms and not taking their physical limits into account resulted in symptom exacerbations. The lack of recognition and validation of symptoms in the absence of a diagnosis or plausible explanation was mentioned as creating difficulties in respecting and resigning to personal physical limits.

"Only, realistically, I sometimes think 'well, but I don't have a problem'. 'There is nothing wrong with me'. Everything is in working order, so I should be able to just do that. Often this is what gets in the way, like 'it's all in my mind'. You know, why not push through? But then I immediately pay the price." (P3, female)

Although many patients mentioned the importance of resigning to physical limits, they described this ongoing process as challenging and often frustrating. By resigning, we mean that patients expressed the need to take their physical limits seriously and anticipate by limiting their activities in order to prevent exacerbations of symptoms. Resigning to limits was experienced as different from accepting their limits, as many kept struggling with the acceptance of their physical limits. They for example encountered new situations as a result of changing environments and life changes over time, again confronting them with their physical limits.

"I still haven't fully embraced it and am not Zen about it. Because, you know, when I see other mothers. (…) Or when Mum plays tag or something. Then I run ten paces. Can't run too long, or I get myself in trouble. That still frustrates me." (P3, female)

Finding a plausible explanation for the experienced symptoms was seen as helpful in accepting their physical limits. In case an explanation was offered and made sense to the patient, it contributed to the understanding of their symptoms and helped them in acceptance of limitations.

Well, that was really good, because then you finally have an explanation for the symptoms. Because she could also explain where this fatigue comes from. And then, well, you adjust your life to it. So I accepted it, that I can just do less. (P2, male)

For peer review only - http://bmjopen.bmj.com/site/about/guidelines.xhtml

Adjusting daily planning

Most patients eventually adjusted their daily planning and routines to their physical limits and capabilities. They mentioned pacing activities and resting effectively as important strategies in gaining control over symptoms and experiencing fewer ups and downs.

"The entire day I keep in mind what I need and want to do. So if I have a birthday tonight, than I take a nap in the afternoon." (P13, male)

In some patients, incorporating mindfulness and relaxation exercises into their daily routine had positive results regarding their experienced symptoms.

Weighing personal needs and learning to say 'no'

Other patients indicated to have gained control by continuously weighing personal needs. Deciding to participate in joyful activities, while they knew it would exacerbate their symptoms, helped some patients to cope. They described to weigh the personal gain and consequences and in some cases decided to consciously overstep their limits, anticipating an exacerbation of symptoms.

"Yes, and also that you know when you are overdoing it and you still choose to do that, knowing that you will be in serious pain the next day. That makes it easier to accept. The harder you fight, the angrier, I think, you will get and the worse your pain will be." (P11, female)

Whereas in some cases the activity was worth overstepping limits, in other cases patients thoughtfully evaluated the activity as having too little value to them. Learning to say no, in such cases, was also experienced as important in gaining control over symptoms.

"And sometimes I think: I am not going to do that. If I am not well and it's not something I really enjoy. No … I evaluate: is it worthwhile, does it do me any good? Is it something I enjoy? If not, I say no. You also need to learn to say 'No'. I didn't do that when this started." (P12, female)

Discussion

Our findings highlight that fluctuations in symptoms, -and in particular the symptom exacerbations that patients describe over the day and week-, are an important element of symptom experience in patients with PPS. It impacts their daily routines and poses various challenges. Patients attributed the experienced worsening of symptoms in particular to overstepping their physical limits and/ or to the impact of negative emotions. Patients described different strategies in gaining control over symptom exacerbations: by resigning to their physical limits, adjusting their daily planning to their limits and capabilities, weighing personal needs and learning to say 'no'.

As far as we are aware, this is the first qualitative study exploring the experiences with fluctuations in symptoms among patients with PPS. A strength of this study was the fact that patients were recruited in different health care settings throughout the Netherlands and that patients varied with regard to diversity of symptoms and demographic and social characteristics. More female patients were interviewed, but numbers were in line with the balance in the cohort from which we selected the patients (75% female). We tried to minimize the impact of our preconceptions, e.g. by our theoretical sampling method in which we included both 'fluctuating' and 'seemingly stable' patients. Although we anticipated differences in experience between these patients, this was in fact not the case. A limitation of our study, is that all interviewed patients experienced (episodes of) severe PPS for longer periods of time, hence our findings may be less applicable to patients experiencing mild symptoms or symptoms of short duration.

For peer review only - http://bmjopen.bmj.com/site/about/guidelines.xhtml

Our findings correspond to some findings from quantitative studies in CFS. Ecologic momentary assessment established that patients experienced difficulties in balancing their activities in response to symptoms. More fatigue related symptoms and pain predicted more activity limitation whereas feeling subjectively well predicted more all-or-nothing behavior, resulting in ups and downs (15). Pacing activities was helpful in preventing fluctuations in symptoms (16). Comparable quantitative studies in a broader sample of patients with PPS are currently lacking. Our findings, however, suggest that dealing with fluctuations -and in particular anticipating on symptom exacerbations- seems to apply to the broader spectrum of PPS.

In our study, resignation to physical limits was mentioned as a strategy to anticipate on and prevent symptom exacerbations. Having a plausible explanation for symptoms was helpful in acceptance of experienced physical limitations. A prior qualitative study described that in particular patients who displayed acceptance of PPS –as opposed to resignation- shifted their focus towards improving their quality of life (26). Resignation and acceptance seem closely related, but the latter implies to be a later stage in a process of change. Acceptance was also an important condition for symptom improvement (27) and facilitated a process of change towards self-compassion and self-care in patients with PPS (28).

While in our study, resigning to limits was described as an important strategy in anticipating on symptom exacerbations and fluctuations in symptoms, Sowińska and Czachowski (29) described how in their population of Polish patients with PPS (MUS) ignoring symptoms or shifting away attention was reported as one of the most successful ways of coping. These differences are interesting. The Polish patients are likely to represent a different selection of patients with PPS: they were all included in the same general practice and visited psychologists and psychiatrist privately. Cultural differences may play a role as well. Multiple studies (26, 30, 31) highlight PPS patients' concerns that it might be 'all in the mind' and how this often brings shame and the feeling of not having 'a legitimate illness'. In our study, patients also struggled with their physical limits in the absence of a 'legitimate illness'. Although symptom exacerbations were attributed to negative emotions by some patients in our study, patients also indicated initial difficulties in accepting the connection. In a recent study on consultations between GPs and patients, symptoms could be attributed to emotions when patients introduced this link themselves. However, when the GP introduced this link it tended to be denied (32). This again underlines the stigma that still pertains on mental distress and its relation to physical health.

Several of our findings may be helpful in the care for patients with PPS. First, our study again underlines the need to take symptoms and their consequences seriously as a health care provider (HCP), also in the absence of an identifiable disease. Patients with PPS face challenges in dealing with fluctuations in symptoms, and more specific in dealing with symptom exacerbations. Secondly, as a HCP, exploring patients' experiences with symptom exacerbations -with attention paid to the experienced impact of physical limits and negative emotions- might be a useful starting point to gain an understanding of what your patient is struggling with on a daily basis and may create a common ground for supportive care to improve wellbeing and provide illness-based interventions and advice.

Our study highlights that fluctuations in symptoms are an important element of the experienced symptoms. More longitudinal research into short-term fluctuations in experienced symptoms in a broad sample of patients with PPS, for example by the experience sampling method (ESM), could provide useful new insights. ESM can reveal how symptom experience relates to implicit patterns of thought, experience and behavior (33). Another valuable area of research could be the different strategies of gaining control over symptom exacerbations and their impact on functional health and wellbeing.

Acknowledgements

For peer review only

We want to thank all the patients that took part in this study for sharing their experiences.

Funding

This work was supported by grants of ZonMw [funding number 839110018] and the Stoffels-Hornstra Foundation. The funding sources had no role in the design, analyses and interpretation of this study.

Author Statement

HB, NC, JCvdW, JD and HvdH developed the study protocol. AdK and ToH provided feedback on the study protocol. HB and EW contributed to the development of the topic guide, collected and analysed the data and interpreted the results. FB analysed the data and interpreted the results. AdK provided feedback on the data collection and analysis. HB drafted the manuscript. All authors read, provided critical revisions and approved the manuscript.

Competing interests

Apart from the institutional grants mentioned above, there are no other potential competing interests to report.

Data sharing statement

No additional data are available.

References

1. Picariello F, Ali S, Moss-Morris R, Chalder T. The most popular terms for medically unexplained symptoms: the views of CFS patients. J Psychosom Res. 2015;78(5):420-6.
2. Marks EM, Hunter MS. Medically Unexplained Symptoms: an acceptable term? Br J Pain. 2015;9(2):109-14.
3. Konnopka A, Schaefert R, Heinrich S, Kaufmann C, Luppa M, Herzog W, et al. Economics of medically unexplained symptoms: a systematic review of the literature. Psychother Psychosom. 2012;81(5):265-75.
4. Rask MT, Ornbol E, Rosendal M, Fink P. Long-term outcome of bodily distress syndrome in primary care: A follow-up study on health care costs, work disability, and self-rated health. Psychosom Med. 2017;79(3):345-57.
5. Nimnuan C, Hotopf M, Wessely S. Medically unexplained symptoms: an epidemiological study in seven specialities. J Psychosom Res. 2001;51(1):361-7.
6. Fink P, Schroder A. One single diagnosis, bodily distress syndrome, succeeded to capture 10 diagnostic categories of functional somatic syndromes and somatoform disorders. J Psychosom Res. 2010;68(5):415-26.
7. Fink P, Toft T, Hansen MS, Ornbol E, Olesen F. Symptoms and syndromes of bodily distress: an exploratory study of 978 internal medical, neurological, and primary care patients. Psychosom Med. 2007;69(1):30-9.
8. Budtz-Lilly A, Schroder A, Rask MT, Fink P, Vestergaard M, Rosendal M. Bodily distress syndrome: A new diagnosis for functional disorders in primary care? BMC Fam Pract. 2015;16:180.
9. Wessely S, White PD. There is only one functional somatic syndrome. Br J Psychiatry. 2004;185:95-6.
10. Jones B. Why we should stop talking about 'medically unexplained symptoms' etc. - the problem of overgeneralisation for research and treatment. J Psychosom Res. 2019;127:109835.
11. Abbi B, Natelson BH. Is chronic fatigue syndrome the same illness as fibromyalgia: evaluating the 'single syndrome' hypothesis. QJM. 2013;106(1):3-9.

For peer review only - http://bmjopen.bmj.com/site/about/guidelines.xhtml

12. White PD. Chronic fatigue syndrome: Is it one discrete syndrome or many? Implications for the "one vs. many" functional somatic syndromes debate. J Psychosom Res. 2010;68(5):455-9.
13. Olde Hartman T, Blankenstein AH, Molenaar B, Bentz van den Berg B, van der Horst HE, Arnold I, et al. [Dutch College of GPs' guideline on medically unexplained physical symptoms] (In Dutch). Huisarts Wet. 2013;56(5):222-30.
14. van der Feltz-Cornelis CM, Swinkels JA, Blankenstein AH, Hoedeman R, Keuter EJ. The Dutch multidisciplinary guideline entitled 'Medically unexplained physical symptoms and somatoform disorder' (In Dutch). Ned Tijdschr Geneeskd. 2011;155(18):A1244..
15. Band R, Barrowclough C, Caldwell K, Emsley R, Wearden A. Activity patterns in response to symptoms in patients being treated for chronic fatigue syndrome: An experience sampling methodology study. Health Psychol. 2017;36(3):264-9.
16. Meeus M, van Eupen I, van Baarle E, De Boeck V, Luyckx A, Kos D, et al. Symptom fluctuations and daily physical activity in patients with chronic fatigue syndrome: a case-control study. Arch Phys Med Rehabil. 2011;92(11):1820-6.
17. Zautra AJ, Fasman R, Parish BP, Davis MC. Daily fatigue in women with osteoarthritis, rheumatoid arthritis, and fibromyalgia. Pain. 2007;128(1-2):128-35.
18. Olde Hartman TC, Borghuis MS, Lucassen PL, van de Laar FA, Speckens AE, van Weel C. Medically unexplained symptoms, somatisation disorder and hypochondriasis: course and prognosis. A systematic review. J Psychosom Res. 2009;66(5):363-77.
19. Claassen-van Dessel N, van der Wouden JC, Hoekstra T, Dekker J, van der Horst HE. The 2-year course of Medically Unexplained Physical Symptoms (MUPS) in terms of symptom severity and functional status: results of the PROSPECTS cohort study. J Psychosom Res. 2018;104:76-87.
20. Kroenke K, Spitzer RL, Williams JB. The PHQ-15: validity of a new measure for evaluating the severity of somatic symptoms. Psychosom Med. 2002;64(2):258-66.
21. VanderZee KI, Sanderman R, Heyink JW, de Haes H. Psychometric qualities of the RAND 36-Item Health Survey 1.0: a multidimensional measure of general health status. Int J Behav Med. 1996;3(2):104-22.
22. van Dessel N, Leone SS, van der Wouden JC, Dekker J, van der Horst HE. The PROSPECTS study: design of a prospective cohort study on prognosis and perpetuating factors of medically unexplained physical symptoms (MUPS). J Psychosom Res. 2014; 76: 200-206.
23. Claassen- van Dessel N, van der Wouden JC, Dekker J, van der Horst HE. Clinical value of DSM IV and DSM 5 criteria for diagnosing the most prevalent somatoform disorders in patients with medically unexplained physical symptoms (MUPS). J Psychosom Res. 2016; 82: 4-10.
24. Braun V and Clarke V. Using thematic analysis in psychology. Qual Res Psychol. 2006(3):77-101.
25. O'Brien BC, Harris IB, Beckman TJ, Reed DA, Cook DA. Standards for reporting qualitative research: a synthesis of recommendations. Acad Med. 2014;89(9):1245-51.
26. Kornelsen J, Atkins C, Brownell K, Woollard R. The meaning of patient experiences of medically unexplained physical symptoms. Qual Health Res. 2016;26(3):367-76.
27. Claassen-van Dessel N Velzeboer FR, van der Wouden JC, den Boer C, Dekker J, van der Horst HE. Patients' perspectives on improvement of medically unexplained physical symptoms: a qualitative analysis. Ment Health Fam Med. 2015;11(02).
28. van Ravesteijn HJ, Suijkerbuijk YB, Langbroek JA, Muskens E, Lucassen PL, van Weel C, et al. Mindfulness-based cognitive therapy (MBCT) for patients with medically unexplained symptoms: process of change. J Psychosom Res. 2014;77(1):27-33.
29. Sowinska A, Czachowski S. Patients' experiences of living with medically unexplained symptoms (MUS): a qualitative study. BMC Fam Pract. 2018;19(1):23.
30. Stone L. Blame, shame and hopelessness: medically unexplained symptoms and the 'heartsink' experience. Aust Fam Physician. 2014;43(4):191-5.
31. Nettleton S, Watt I, O'Malley L, Duffey P. Understanding the narratives of people who live with medically unexplained illness. Patient education and counseling. 2005;56(2):205-10.

For peer review only - http://bmjopen.bmj.com/site/about/guidelines.xhtml

32. Bekhuis E, Gol J, Burton C, Rosmalen J. Patients' descriptions of the relation between physical symptoms and negative emotions: a qualitative analysis of primary care consultations. Br J Gen Pract. 2020;70(691):e78-e85.
33. van Os J, Verhagen S, Marsman A, Peeters F, Bak M, Marcelis M, et al. The experience sampling method as an mHealth tool to support self-monitoring, self-insight, and personalized health care in clinical practice. Depress Anxiety. 2017;34(6):481-93.

For peer review only

For peer review only - http://bmjopen.bmj.com/site/about/guidelines.xhtml

Reporting checklist for qualitative study.

Based on the SRQR guidelines.

Instructions to authors

Complete this checklist by entering the page numbers from your manuscript where readers will find each of the items listed below.

Your article may not currently address all the items on the checklist. Please modify your text to include the missing information. If you are certain that an item does not apply, please write "n/a" and provide a short explanation.

Upload your completed checklist as an extra file when you submit to a journal.

In your methods section, say that you used the SRQRreporting guidelines, and cite them as:

O'Brien BC, Harris IB, Beckman TJ, Reed DA, Cook DA. Standards for reporting qualitative research: a synthesis of recommendations. Acad Med. 2014;89(9):1245-1251.

		Reporting Item	Page Number
**Title**			
	#1	Concise description of the nature and topic of the study identifying the study as qualitative or indicating the approach (e.g. ethnography, grounded theory) or data collection methods (e.g. interview, focus group) is recommended	1
**Abstract**			
	#2	Summary of the key elements of the study using the abstract format of the intended publication; typically includes background, purpose, methods, results and conclusions	2
**Introduction**			
Problem formulation	#3	Description and significance of the problem / phenomenon studied: review of relevant theory and empirical work; problem statement	4

For peer review only

Purpose or research question	#4	Purpose of the study and specific objectives or questions	4
**Methods**			
Qualitative approach and research paradigm	#5	Qualitative approach (e.g. ethnography, grounded theory, case study, phenomenolgy, narrative research) and guiding theory if appropriate; identifying the research paradigm (e.g. postpositivist, constructivist / interpretivist) is also recommended; rationale. The rationale should briefly discuss the justification for choosing that theory, approach, method or technique rather than other options available; the assumptions and limitations implicit in those choices and how those choices influence study conclusions and transferability. As appropriate the rationale for several items might be discussed together.	4-7
Researcher characteristics and reflexivity	#6	Researchers' characteristics that may influence the research, including personal attributes, qualifications / experience, relationship with participants, assumptions and / or presuppositions; potential or actual interaction between researchers' characteristics and the research questions, approach, methods, results and / or transferability	7
Context	#7	Setting / site and salient contextual factors; rationale	4,6,7
Sampling strategy	#8	How and why research participants, documents, or events were selected; criteria for deciding when no further sampling was necessary (e.g. sampling saturation); rationale	4,5
Ethical issues pertaining to human subjects	#9	Documentation of approval by an appropriate ethics review board and participant consent, or explanation for lack thereof; other confidentiality and data security issues	4
Data collection methods	#10	Types of data collected; details of data collection procedures including (as appropriate) start and stop dates of data collection and analysis, iterative	7

For peer review only

		process, triangulation of sources / methods, and modification of procedures in response to evolving study findings; rationale	
Data collection instruments and technologies	#11	Description of instruments (e.g. interview guides, questionnaires) and devices (e.g. audio recorders) used for data collection; if / how the instruments(s) changed over the course of the study	7
Units of study	#12	Number and relevant characteristics of participants, documents, or events included in the study; level of participation (could be reported in results)	5,6
Data processing	#13	Methods for processing data prior to and during analysis, including transcription, data entry, data management and security, verification of data integrity, data coding, and anonymisation / deidentification of excerpts	7
Data analysis	#14	Process by which inferences, themes, etc. were identified and developed, including the researchers involved in data analysis; usually references a specific paradigm or approach; rationale	7
Techniques to enhance trustworthiness	#15	Techniques to enhance trustworthiness and credibility of data analysis (e.g. member checking, audit trail, triangulation); rationale	7
**Results/findings**			
Syntheses and interpretation	#16	Main findings (e.g. interpretations, inferences, and themes); might include development of a theory or model, or integration with prior research or theory	8,9,10,11
Links to empirical data	#17	Evidence (e.g. quotes, field notes, text excerpts, photographs) to substantiate analytic findings	8,9,10,11
**Discussion**			
Intergration with prior work, implications, transferability and contribution(s) to the field	#18	Short summary of main findings; explanation of how findings and conclusions connect to, support, elaborate on, or challenge conclusions of earlier scholarship; discussion of scope of application /	11,12

For peer review only

		generalizability; identification of unique contributions(s) to scholarship in a discipline or field	
Limitations	#19	Trustworthiness and limitations of findings	11,12
**Other**			
Conflicts of interest	#20	Potential sources of influence of perceived influence on study conduct and conclusions; how these were managed	11
Funding	#21	Sources of funding and other support; role of funders in data collection, interpretation and reporting	13

The SRQR checklist is distributed with permission of Wolters Kluwer © 2014 by the Association of American Medical Colleges. This checklist was completed on 15. November 2019 using https://www.goodreports.org/, a tool made by the EQUATOR Network in collaboration with Penelope.ai

For peer review only
